# Responses to larval herbivory in the phenylpropanoid pathway of *Ulmus minor* are boosted by prior insect egg deposition

**DOI:** 10.1007/s00425-021-03803-0

**Published:** 2021-12-08

**Authors:** Johanna Schott, Benjamin Fuchs, Christoph Böttcher, Monika Hilker

**Affiliations:** 1 Department of Applied Zoology/Animal Ecology, Dahlem Centre of Plant Sciences, Freie Universität Berlin, Haderslebener Str. 9, 12163 Berlin, Germany; 2 Institute for Ecological Chemistry, Plant Analysis and Stored Product Protection, Julius Kühn Institute (JKI)-Federal Research Centre for Cultivated Plants, Königin-Luise-Str. 19, 14195 Berlin, Germany; 3 Present Address: Biodiversity Unit, University of Turku, 20014 Turku, Finland

**Keywords:** Flavonoids, Insect herbivory, Metabolomics, Plant defence, Priming, Salicylic acid

## Abstract

**Main conclusion:**

Elms, which have received insect eggs as a ‘warning’ of larval herbivory, enhance their anti-herbivore defences by accumulating salicylic acid and amplifying phenylpropanoid-related transcriptional and metabolic responses to hatching larvae.

**Abstract:**

Plant responses to insect eggs can result in intensified defences against hatching larvae. In annual plants, this egg-mediated effect is known to be associated with changes in leaf phenylpropanoid levels. However, little is known about how trees—long-living, perennial plants—improve their egg-mediated, anti-herbivore defences. The role of phytohormones and the phenylpropanoid pathway in egg-primed anti-herbivore defences of a tree species has until now been left unexplored. Using targeted and untargeted metabolome analyses we studied how the phenylpropanoid pathway of *Ulmus minor* responds to egg-laying by the elm leaf beetle and subsequent larval feeding. We found that when compared to untreated leaves, kaempferol and quercetin concentrations increased in feeding-damaged leaves with prior egg deposition, but not in feeding-damaged leaves without eggs. PCR analyses revealed that prior insect egg deposition intensified feeding-induced expression of *phenylalanine ammonia lyase* (*PAL*), encoding the gateway enzyme of the phenylpropanoid pathway*.* Salicylic acid (SA) concentrations were higher in egg-treated, feeding-damaged leaves than in egg-free, feeding-damaged leaves, but SA levels did not increase in response to egg deposition alone—in contrast to observations made of *Arabidopsis thaliana*. Our results indicate that prior egg deposition induces a SA-mediated response in elms to feeding damage. Furthermore, egg deposition boosts phenylpropanoid biosynthesis in subsequently feeding-damaged leaves by enhanced *PAL* expression, which results in the accumulation of phenylpropanoid derivatives. As such, the elm tree shows similar, yet distinct, responses to insect eggs and larval feeding as the annual model plant *A. thaliana.*

**Supplementary Information:**

The online version contains supplementary material available at 10.1007/s00425-021-03803-0.

## Introduction

Trees are a rich food source for many herbivorous insects. Herbivory may threaten forest ecosystems by massive defoliation and the transmission of serious diseases (Boyd et al. 2013). Trees have evolved a plethora of chemical and physical adaptations, which improve their survival in spite of frequent and severe stresses induced by insect infestations (Boeckler et al. 2011; Büchel et al. 2016; Caldwell et al. 2016). Stress-induced resistance in plants is an ‘on-demand’ strategy that invests resources in defences as they are needed (Wu and Baldwin 2010; Karban 2011; Stam et al. 2014; Turlings and Erb 2018). Several studies have shown that plants can even improve their inducible stress resistance by responding to environmental cues for impending stress; in this way, plants are able to ‘get ready’ and primed for mobilising their stress responses (Frost et al. 2008; Conrath et al. 2015; Mauch-Mani et al. 2017).

Environmental cues priming anti-herbivore defences include the first feeding damage prior to subsequent damage, leaf volatiles emitted by damaged neighbouring plants, and insect egg depositions that warn of impending larval feeding damage (Hilker et al. 2016, and references therein). Trees responding to these cues have been shown to improve their anti-herbivore defences against subsequently occurring insect herbivory (Haukioja 1991; Tscharntke et al. 2001; Frost et al. 2008; Beyaert et al. 2012; Austel et al. 2016; Li and Blande 2017).

However, the underlying mechanisms for priming inducible defences using herbivory-indicating cues have so far mainly been studied in annual plant species (Reymond 2013; Hilker and Fatouros 2016; Wilkinson et al. 2019). The feeding-induced phytohormonal and metabolic responses of several annual plant species that have been primed by insect egg deposition prior to larval herbivory differ from the feeding-induced responses of egg-free (non-primed) plants. These responses have been studied especially in Brassicaceae and Solanaceae. Phytohormonal analyses have revealed that egg-primed, feeding-induced brassicaceous plants show increased concentrations of salicylic acid (SA) (Bonnet et al. 2017; Lortzing et al. 2019). Egg-primed solanaceous plant species were found to respond to damage with higher concentrations of jasmonic acid (JA) (Kim et al. 2012) or enhanced transcription of a JA-responsive transcription factor (Bandoly et al. 2015). Egg deposition also affects the feeding-induced response of the phenylpropanoid pathway in both brassicaceous and solanaceous plants. Egg-primed, feeding-induced leaves of these plant taxa show higher levels of certain phenylpropanoid derivatives than non-primed ones after feeding damage (Bandoly et al. 2015; Lortzing et al. 2019) and enhanced expression of genes involved in the phenylpropanoid pathway (Geuss et al. 2018).

Previous studies indicate that phenylpropanoids also play a role in priming anti-herbivore defences in the field elm, *Ulmus minor* (Austel et al. 2016; Altmann et al. 2018)*.* Elm responses to egg deposition by the elm leaf beetle *Xanthogaleruca luteola* enhance the tree’s defences against larvae of this beetle species. Larvae feeding upon previously egg-laden leaves were shown to suffer higher mortality than larvae starting their development on egg-free leaves (Austel et al. 2016). Larvae that began their development on an egg-treated tree faced higher concentrations of a flavonoid glycoside, kaempferol-3-*O*-robinoside-7-*O*-rhamnoside, after an 8-day feeding period. Furthermore, application of high concentrations of this kaempferol glycoside onto (egg-free) elm leaves resulted in higher larval mortality (Austel et al. 2016). These results indicate the importance of this type of flavonoids for improved, egg-primed responses of elms to larval herbivory.

Flavonoids are late products of the phenylpropanoid pathway. The enzyme phenylalanine ammonia lyase (PAL) catalyses a crucial step at the beginning of the phenylpropanoid pathway, the deamination of phenylalanine. The resulting product, *trans-*cinnamic acid, is hydroxylated to 4-coumarate, which is further converted to 4-coumaryl-CoA; this step is catalysed by the enzyme 4-coumarate CoA ligase (4CL). From 4-coumaryl-CoA, the path branches in several directions. The reaction of shikimate *O*-hydroxycinnamoyl-transferase (HCT) with 4-coumaryl-CoA as substrate continues to lignin-building units, which can be further modified by caffeic acid 3-*O*-methyltransferase (COMT) and cinnamyl alcohol dehydrogenase (CAD). In addition to the ‘lignin branch’, the phenylpropanoid pathway also deviates from 4-coumaryl-CoA to the biosynthesis of flavonoids. The flavonoid biosynthesis leading to kaempferol and quercetin derivatives involves, among other enzymes, F3H (flavanone 3-hydroxylase), FLS (flavonol synthase), and F3′H (flavonoid 3′-monooxygenase). Further downstream of the flavonoid branch, anthocyanin derivatives and catechins are produced; it is here that the enzyme leucoanthocyanidin dioxygenase (ANS) is involved (Vogt 2010; Tohge et al. 2017).

Several flavonoids, among them kaempferol and quercetin, glycosylated at different positions by various sugars, have been detected in elm leaves (Santamour 1972; Bate-Smith and Richens 1973; Martín et al. 2013). A previous comparative RNA-seq analysis of egg-treated and egg-free elm leaves after 6 h of feeding damage by elm leaf beetle larvae also pointed to a role of phenylpropanoids in egg-mediated priming of the elm’s anti-herbivore defences (Altmann et al. 2018). The RNA-seq analysis identified enrichments in gene ontology (GO) terms related to cell wall organisation and phenylpropanoid metabolic and biosynthetic processes (Altmann et al. 2018).

The results of our earlier studies on elm responses to elm leaf beetle infestation prompted us to investigate further how egg-mediated priming of anti-herbivore defences in elm is processed. The aim of the present study was to elucidate how the phenylpropanoid pathway of egg-primed elm leaves responds to feeding damage by elm leaf beetle larvae. Furthermore, we aimed to determine which phytohormonal changes are involved in egg-priming of the elm’s anti-herbivore defences. We addressed the following questions: (1) How does the beetle’s egg deposition affect levels of phytohormones in elm leaves subsequently damaged by larval feeding? For this, we analysed levels of SA, JA, JA-isoleucine (JA-Ile) and abscisic acid (ABA). (2) How do transcript levels of genes involved in the phenylpropanoid pathway change in egg-free and egg-primed leaves after the onset of larval feeding? To address this question, we studied transcript levels of the aforementioned (homologues of) genes encoding enzymes catalysing phenylpropanoid biosynthesis. (3) Do phenylpropanoid concentrations in egg-primed leaves increase after the onset of larval feeding?

With respect to questions (1) and (2), we took into account that changes of phytohormone levels and gene expression are known to vary rapidly following egg deposition and/or feeding (e.g., Altmann et al. 2018; Farmer et al. 2020). We analysed these parameters 6 h and 24 h after the onset of larval feeding upon egg-free or previously egg-laden leaves. These time points were chosen since the elm RNA-seq analyses by Altmann et al. (2018) showed differential gene expression in egg-primed leaves early after larvae started feeding. Furthermore, we were interested in the spatial distribution of the phytohormonal and transcriptional responses to elm leaf beetle eggs and feeding. Therefore, we analysed both locally treated leaves and leaves adjacent to them. With respect to question (3), we focussed our analyses on locally treated leaves harvested 24 h after the onset of feeding because our analyses of phytohormone levels and gene expression revealed especially strong local effects at this time point.

## Materials and methods

### Plants and insects

Seed-grown, 1-year-old elm trees (*Ulmus minor* Miller) were purchased from Baumschule Appel (Waldsieversdorf, Germany) and potted in 5 L pots with potting soil Classic T (Einheitserde^®^, Uetersen, Germany) mixed with 5% vermiculite. The trees were kept in a greenhouse under long day conditions. In addition to daylight, trees were supplemented with light (EYE IWASAKI MT 400W/DL, Iwasaki electric Co. Ltd., Tokyo, Japan) for 16 h a day. The 1-year-old trees used in our experiments were about 0.80–1.20 m tall and had grown up to ten branches deviating from the main axis. Each branch showed up to 18 fully developed leaves.

Elm leaf beetles (*Xanthogaleruca luteola* Müller) were collected from a natural population around Montpellier, France, where they occur in high densities. In the laboratory, the beetles were kept in microperforated polypropylene bags on twigs of potted elm trees at room temperature (22–25 °C, 16:8 h light/dark with 625–800 lx, 65–75% relative humidity). Inside these bags, beetles fed, mated and laid eggs upon leaves. Once several egg clutches were laid per twig, beetles were transferred to a new twig. Twigs with freshly hatched larvae were placed into plastic boxes covered with a gauze lid. The larvae were provided with fresh elm twigs three times per week until they pupated.

### General experimental design and conditions

The experiments were conducted in our greenhouse between May and August. Elm trees were subjected to the following three treatments: (i) standardised egg deposition (E), (ii) larval feeding (F) upon egg-free leaves, and (iii) standardised egg deposition plus larval feeding (EF); this latter treatment was used to study how egg deposition affects the responses of leaves to subsequent larval feeding damage. To complete a full-factorial experimental design, we left elm trees untreated as controls (C).

**Fig. 1:**
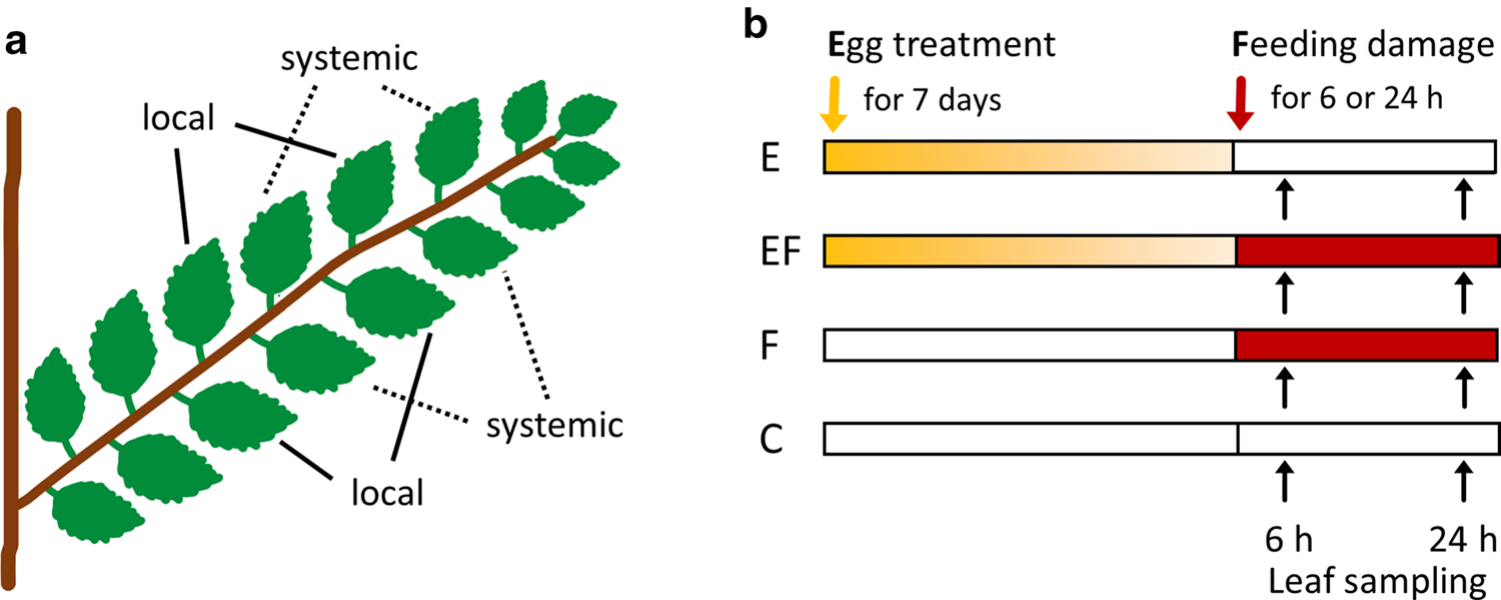
Experimental design. **a** Position of locally treated *Ulmus minor* leaves (= local leaves) and adjacent leaves left untreated (= systemic leaves). We investigated local and systemic leaves. **b** Local leaves were exposed to an egg treatment and feeding damage by larvae of the elm leaf beetle *Xanthogaleruca luteola.* Treatments: E = egg treatment, EF = egg treatment and, 7 days later, larval feeding damage, F = feeding damage, C = untreated control. Yellow arrow: time point for egg treatment; red arrow: start of feeding damage by neonate larvae; duration of egg treatment: 7 days (i.e., natural egg incubation time for larvae to hatch); duration of larval feeding: 6 h or 24 h. Black arrows: leaf material was sampled 6 h or 24 h after the onset of feeding and at equivalent time points in C and E leaves

One branch per tree was chosen for the treatments. All treatments were applied to four leaves per tree in the following manner: counting from the branching point, the first four leaves of a branch remained untreated and were not considered for sampling. They were followed by two treated, then two untreated, and then two treated leaves, ending with two untreated leaves. The sampled treated leaves are referred to as ‘local’ leaves, while the sampled untreated leaves are referred to as ‘systemic’ leaves (Fig. 1a).

Local and systemic leaves were harvested at consistent time points after treatment. Leaves subjected to the F and EF treatments were harvested 6 h or 24 h after the onset of larval feeding. Leaves from control trees and trees subjected to the E treatment were harvested at equivalent time points, i.e., after a 7-day E treatment period plus 6 h or 24 h (Fig. 1b). Harvesting E samples at these time points allowed us to determine whether an elm’s response to the egg treatment was maintained during the natural egg incubation time, which lasts for 7 days, until larvae hatch from eggs. Immediately after harvesting, leaves from all treatments (C, E, F, EF) were stored in liquid nitrogen.

Each treatment was replicated ten times for each of the two harvest time points (i.e., there were 80 treated plants in total). Within each replicate, trees were treated on the same day and were of the same age and of comparable size. Treatments were always applied between 10.00 a.m. and 11.00 a.m., to avoid possible variation in the parameters analysed due to time of day.

### Plant treatments: exposure to egg deposition and feeding

To standardise the number, time, and site of egg depositions for the E and EF treatments, we mimicked natural egg deposition using the protocol described by Austel et al. (2016) and Altmann et al. (2018). Briefly, a small piece (15 mm × 1 mm) of the abaxial leaf epidermis was gently removed with a scalpel, thus mimicking how a female elm leaf beetle removes leaf tissue with her mandibles prior to egg deposition at the oviposition site. This treatment was immediately followed by application of oviduct secretion from a freshly killed, gravid female beetle. This secretion envelops the eggs and is in immediate contact with the leaf. The *oviductus communis* of a female provided sufficient secretion for two standardised egg treatments. Previous studies have shown that treatment of leaves with oviduct secretion, as described here, elicits a plant response similar to that observed after natural egg deposition (Meiners and Hilker 2000; Austel et al. 2016); we refer to these as ‘egg-treated’ leaves.

For the feeding stimulus in F and EF treatments, we used neonate larvae from our rearing. Five newly hatched larvae were placed with a smooth brush onto each egg-treated leaf of an EF tree and onto the corresponding leaves of a F tree. This was carried out for the EF trees 7 days after treating the leaves with standardised egg deposition, and at the equivalent time for the F trees. The neonate larvae were restricted to feeding at the correct leaves by caging them in a plastic clip cage, which was gently fixed to the leaf. Leaves of C and E trees received (empty) clip cages at the same time points and positions on the branch.

### Determination of phytohormone concentrations

To determine how elm leaf beetle egg deposition and larval feeding damage affected concentrations of the phytohormones SA, JA, JA-Ile, and ABA, phytohormone concentrations were determined 6 h and 24 h after onset of feeding in F and EF leaves and at the corresponding time points in C and E leaves. Both locally treated and systemic leaves were analysed. We used leaf powder aliquots of 80–100 mg (fresh weight) each. The extraction procedure followed the protocol described by Wang et al. (2007). We added 1 mL ethyl acetate to each sample, together with an internal standard mix of the deuterated phytohormones d4-salicylic acid, d6-abscisic acid (both from OlChemIm Ltd., Olomouc, Czech Republic), d6-jasmonic acid and d6-jasmonyl-l-isoleucine (both from HPC Standards GmbH, Cunnersdorf, Germany). Samples were homogenised in a FastPrep instrument (MP Biomedicals, Solon, USA), followed by centrifugation. Afterwards, supernatants were transferred into new 2 mL tubes. Extraction was repeated using 1 mL ethyl acetate, and supernatants were combined, followed by evaporation to a honey-like viscosity (approx. 10 µL volume) (Eppendorf Concentrator 5301, Eppendorf AG, Hamburg, Deutschland). Extracts were re-dissolved in 400 µL 70% methanol with 0.1% formic acid and stored at − 20 °C until analysis. Phytohormone contents were analysed from 7 µL solution injected into an UPLC–ESI–MS/MS on a Synapt G2-S HDMS (Waters^®^, Milford, MA, USA). The detailed extraction protocol and UPLC conditions are described in Supplementary Information: protocols/phytohormone extraction and measurement.

### qRT-PCR analysis

To determine how elm leaf beetle egg deposition and larval feeding affect the expression levels of candidate elm genes involved in the phenylpropanoid pathway, we conducted qRT-PCR analyses using local and systemic leaves from the differently treated trees. Samples were harvested 6 h and 24 h after the onset of feeding from locally treated F and EF leaves and at the corresponding time points from C and E leaves. Samples from systemic F and EF leaves were harvested only after a 24 h feeding period and at the corresponding time from C and E leaves.

The plant material was ground under liquid nitrogen to a fine leaf powder. Total RNA from 50 to 60 mg leaf powder (fresh weight) was extracted according to a chloroform-based protocol modified from Altmann et al. (2018) and Ikoma et al. (1996) (for further details, see Supplementary Information: protocols/RNA extraction). Any residual DNA was removed using DNase with the aid of DNA-free™ Kit (Thermo Fisher Scientific, Waltham, MA, USA). RNA concentration and purity were determined spectrophotometrically, and RNA integrity was verified by gel electrophoresis on 1.8% agarose gels.

First-strand cDNA was synthesised from 1 μg total RNA with the RevertAid™ RT Reverse Transcription Kit (Thermo Fisher Scientific). The protocol was modified to use oligo-dT and random hexamers to facilitate reverse transcription. Quantitative real time PCR (qRT-PCR) with 10 µL reactions was performed in technical triplicates with 10 ng cDNA and 5 µL Power SYBR^©^ Green Master Mix (Thermo Fisher Scientific) on a Stratagene Mx3005P Real-Time PCR System (Agilent Technologies Inc., Santa Clara, CA, USA) with a thermal profile of 1 × (95 °C for 10 min), 45 × (95 °C for 20 s and 60 °C for 60 s), followed by a melting curve analysis (55–95 °C).

Based on screening the *U. minor* RNA-seq data set provided by Altmann et al. (2018), we analysed sequences, which are homologues of genes known to be involved in the phenylpropanoid pathway. Primers were successfully designed using Clone Manager Suite 7 (Sci Ed Software, Westminster, CO, USA) for the sequences listed in Table S1. Each primer pair was tested for amplification efficiency and specificity by melting curve analysis and gel electrophoresis on a 2.7% agarose gel. The sequences analysed are putatively encoding the following enzymes: PAL, 4CL, HCT, COMT, CAD, FLS/F3H, F3′H and ANS. The *FLS/F3H* sequence analysed here is based on the respective sequence published by Perdiguero et al. (2015), who conducted RNA-seq analyses of three *U. minor* genotypes. Perdiguero et al. (2015) annotated the *FLS/F3H* sequence as flavonol synthase/flavanone-3-hydroxylase because it could not be determined unambiguously which of the two enzymes is encoded by this sequence. We decided to analyse transcript levels of the *FLS/F3H* sequence described by Perdiguero as (i) both enzymes catalyse biosynthesis steps between *p*-coumaroyl-CoA and kaempferol, and (ii) kaempferol derivatives are believed to play a role in elm defences against herbivory (Austel et al. 2016).

When quantifying transcript levels of the putative phenylpropanoid biosynthesis genes, we used sequences homologous to *SAND family gene*, *UBQ* (*polyubiquitin*), and *Splicing factor3B subunit 5-like* for normalisation. These reference genes showed treatment-independent, stable expression in a previous study, which sequenced and analysed the transcriptome of elm exposed to *X. luteola* egg deposition and larval feeding (Altmann et al. 2018). Primer sequences of the reference genes are included in Table S2. For quantification of transcripts, we calculated a reference gene index by determining the geometric mean of the expression levels of the three reference genes (Vandesompele et al. 2002). The relative expression levels of each sequence mentioned in Table S1 were calculated by relating the determined 2^−ΔCt^ values to the reference index according to a modified protocol of Livak and Schmittgen (2001).

### Preparation of leaf extracts for metabolite analysis

To determine changes in the levels of semipolar leaf metabolites in response to the study treatments, methanolic leaf extracts were prepared for HPLC–DAD and UHPLC–ESI–QTOFMS.

We prepared crude extracts from leaves subjected to the different treatments. Leaf metabolites were exclusively analysed in locally treated leaves harvested 24 h after the onset of larval feeding in F and EF leaves and in C and E leaves at the equivalent time. We focussed the metabolite analysis on these leaves because our earlier qRT-PCR and phytohormone analyses indicated treatment effects in these leaves in particular.

To obtain a crude leaf extract, an aliquot (50 mg) of finely ground leaf powder was transferred under cooling with liquid nitrogen into a 2 mL Eppendorf tube. The tube was placed in a cooling rack (CoolRack, Corning Inc., Corning, NY, USA) in liquid nitrogen. We added 750 µL 80% methanol to the powder in the tube; prior to this, the methanol had been cooled to − 80 °C. The methanol added contained 4-chlorophenylalanine, umbelliferone, aspartame, phloridzin and biochanin A, each at 2 µM, as internal standards. The mixture was vortexed for 10 s and returned to the cooling rack for 5 min. Vortexing for 10 s and cooling for 5 min were repeated for 30 min, until samples were fully thawed. All subsequent steps were performed at room temperature. The vortexed sample was ultrasonicated for 15 min. After centrifugation for 10 min at 18,213*g* (Eppendorf 5437 R centrifuge), 650 µL of the resulting supernatant were transferred into a 2 mL reaction tube (Eppendorf). The remaining residue was extracted a second time with another 750 µL 80% aqueous methanol by vortexing for 15 min, followed by 15 min ultrasonication and 10 min centrifugation at 18,213*g*. Both supernatants were combined and provided 1300 µL of leaf crude extract. The crude extracts were further processed for leaf metabolite analyses, as described below.

### Analysis of total kaempferol and quercetin

HPLC–DAD was used to absolutely quantify the levels of two major flavonoids in the differently treated elm leaves. We focussed our analyses on kaempferol and its 3′-hydroxy derivative, quercetin, because a study by Austel et al. (2016) showed that a kaempferol derivative is detrimental to elm leaf beetle larvae. To determine the total concentration of the flavonol core structures, rather than the concentration of each of their various glycosides, we subjected the crude elm leaf extract to acidic hydrolysis according to a modified protocol from Hertog et al. (1992) and Mattila et al. (2000). A volume of 700 µL leaf crude extract was transferred into a 2 mL centrifuge tube and evaporated to dryness under reduced pressure at 40 °C (Eppendorf Concentrator 5301). The remaining residue was suspended in 350 µL 1.2 M HCl, sonicated for 10 min and incubated for 1 h at 80 °C under constant shaking. After cooling to room temperature, 700 µL ethyl acetate were added, and the mixture was vortex-mixed for 10 min. After centrifugation for 10 min at 8000*g* the organic phase was transferred into a 2 mL reaction tube. The remaining aqueous phase was extracted with another 700 µL ethyl acetate. The combined organic extracts were evaporated to dryness under reduced pressure at 40 °C. The resulting residue was dissolved in 200 µL 70% methanol and subjected to HPLC–DAD (for details, see Supplementary Information: protocols/HPLC–DAD settings for analyses of flavonol aglycones). Quantification was carried out by external calibration curves with kaempferol and quercetin (Sigma Aldrich Corp., St. Louis, MO, USA).

### Phenylpropanoid metabolite profiling

Ultra-high performance liquid chromatography/electrospray ionisation–quadrupole time-of-flight mass spectrometry (UHPLC/ESI–QTOFMS) was used to annotate and quantify levels of flavonol glycosides and several other phenylpropanoid derivatives in treated leaves relative to the levels in untreated control leaves.

For this quantitative analysis of metabolites, crude leaf extract (200 µL) was transferred into a 2 mL centrifuge tube and evaporated to dryness under reduced pressure at 40 °C. We added 200 µL 50% methanol acidified with 0.1% (v/v) formic acid to the remaining residue. The mixture was ultrasonicated for 10 min and centrifuged for 10 min at 18,213*g*. The resulting supernatant (1 µL of each type of leaf extract) was subjected to UHPLC/ESI–QTOFMS analysis operated in negative ion mode. Prior to non-targeted analysis, a quality control was performed (for details, see Supplementary Information: protocols/UHPLC/ESI–QTOFMS settings for untargeted analyses and quality control). Raw data were analysed using the XCMS algorithm (non-targeted analysis) and MassHunter Qualitative and Quantitative Analysis software (Version B.07.00, Agilent Technologies Inc.) (targeted analysis). The parameter settings of feature detection, alignment, normalisation, and filtering are described in Supplementary Information: protocols/pre-processing of untargeted UHPLC/ESI–QTOFMS data of semipolar compounds.

The filtered peak area matrix (1395 features in all samples) was explored to identify quantitatively differential features. To this end, comparisons between the means of the log_2_-transformed peak areas of the following sample class pairs were performed using Welch’s test, uncorrected for multiple testing: (i) C vs. E, (ii) C vs. F, (iii) C vs. EF, (iv) F vs. EF. With a significance threshold of *P* < 0.05 and an absolute difference threshold ∣Δ mean∣ ≥ 1, a total of 124 features were found, which showed different peak areas in at least one of the four pairwise comparisons.

For metabolite annotation, these 124 differential features were grouped by retention time and assigned to putative compound mass spectra based on the similarity of their elution profiles. After identifying pseudo-molecular, adduct and cluster ions within a putative compound mass spectrum, accurate mass collision-induced dissociation (CID) mass spectra were acquired by UHPLC/ESI–QTOFMS in targeted MS/MS mode using scheduled precursor ion lists. CID spectra were compared with those in databases or were manually interpreted. Putative annotations were validated by comparison with chromatographic and mass spectral data of authentic compounds, wherever possible (for details, see Supplementary Information: protocols/compound annotation by UHPLC/ESI–QTOFMS in targeted MS/MS mode). For comprehensive annotation of flavonol glycosides, an all-ion fragmentation approach was used. For respective chromatograms see Fig. S1 (*O*-glycosylated flavonols) and Fig. S2 (flavan-3-ols and derived dimeric and trimeric proanthocyanidins).

For final statistical evaluations and visualisation of quantitative differences, all annotated metabolites detected by UHPLC/ESI–QTOFMS were quantified again via a manual process using MassHunter Quantitative Analysis software; we listed *m/z* and the retention times of respective quantifier ions in Table S3. The results of this quantification were set relative to the quantities detected in untreated control leaves and were normalised to leaf fresh weight.

### Statistics

All statistical analyses were performed with the software “R”, version 4.0.2 (R Core Team 2020), using the packages car (version 3.0-9, Fox and Weisberg 2018), ggplot2 (version 3.3.2, Wickham 2016), plyr (version 1.8.6, Wickham 2011), and multcomp (version 1.4-13, Hothorn et al. 2008). For untargeted metabolome analysis, we used XCMS (Smith et al. 2006), and for the heatmap we used ComplexHeatmap (version 2.4.3, Gu et al. 2016, http://bioconductor.org/biocLite.R).

Data were checked for normal distribution using Q–Q plots. Homogeneity of variances was tested with Levene’s test. Data not normally distributed were log_2_-transformed and analysed again. Extreme outliers (values above Q3 + 3 × IQR, or below Q1 − 3 × IQR) were not included in the statistical evaluation of data obtained from analyses of concentrations of phytohormones, transcript levels, or levels of hydrolysed metabolites. Normally distributed data were evaluated by one-way ANOVA and post-hoc general linear hypothesis test (glht) with Tukey contrasts. Data not normally distributed even after log_2_‐transformation were analysed using the Kruskal–Wallis test and post-hoc pairwise comparisons using Benjamini–Hochberg-corrected Wilcoxon rank-sum tests.

## Results

### Effects of egg deposition and larval feeding on elm phytohormone levels

**Fig. 2:**
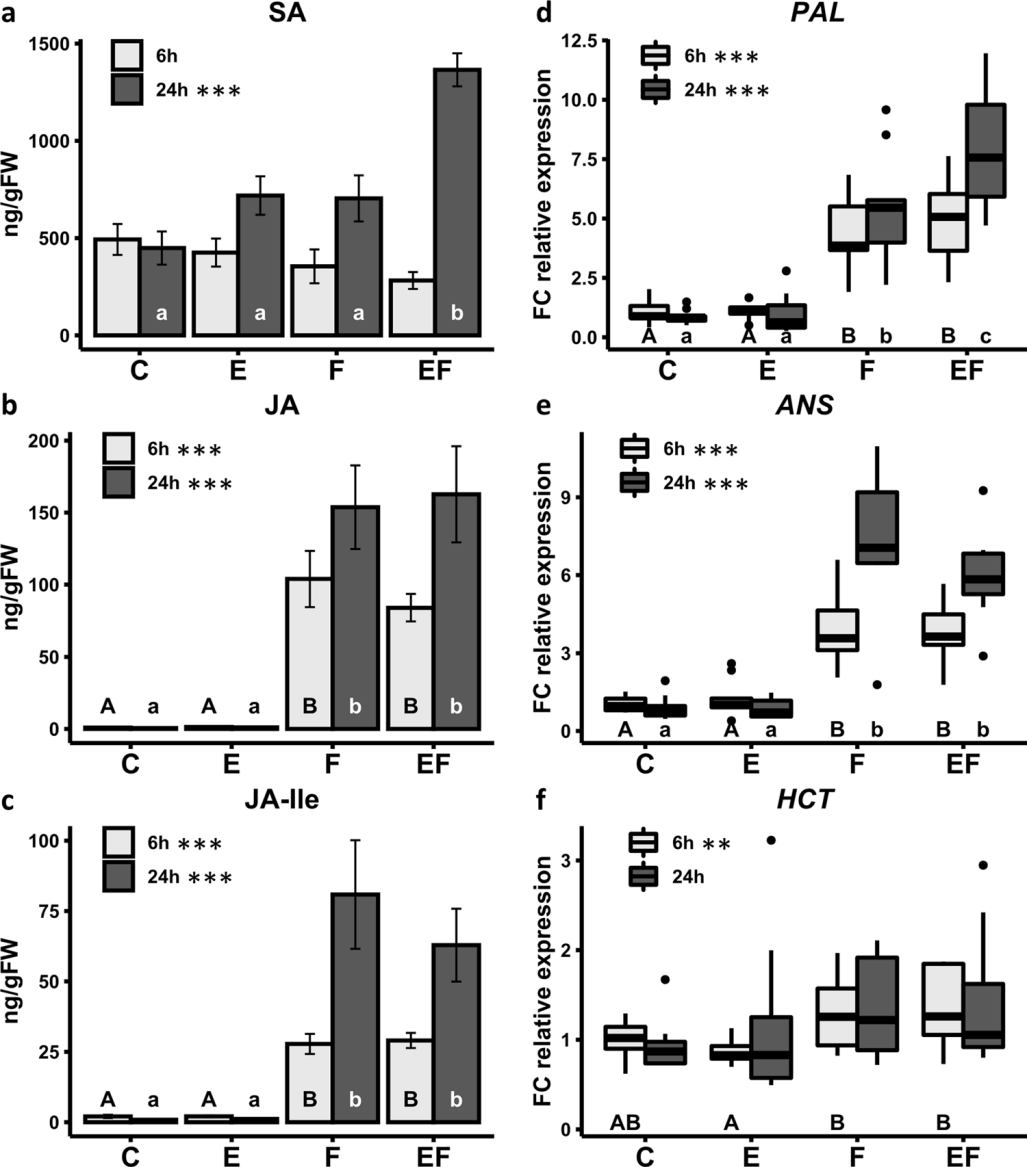
Effects of elm leaf beetle egg deposition and larval feeding on phytohormone concentrations and expression of *Ulmus minor* genes involved in phenylpropanoid biosynthesis. Concentrations of **a** salicylic acid (SA), **b** jasmonic acid (JA), and **c** jasmonic acid-isoleucine (JA-Ile), as well as expression of **d** *PAL* (*phenylalanine ammonia lyase*) **e** *ANS* (*leucoanthocyanidin dioxygenase*), **f** *HCT* (*shikimate* O-*hydroxycinnamoyl-transferase*) were measured in locally treated leaves after 6 h (grey bars) or 24 h (black bars) of larval feeding, and at equivalent time points for treatments without larval feeding. C = untreated control leaves, E = egg-treated leaves, F = feeding-damaged leaves, EF = egg-treated, feeding-damaged leaves. **a**–**c** Bars represent means ± SE of *n* = 9–10 samples. One-way ANOVA was performed separately for log-transformed data from 6 and 24 h samples. In cases of a significant result (*P < 0.05, **P < 0.01, ***P < 0.001), Tukey test with single-step adjusted *P* values was carried out as a post-hoc test. **d**–**f** Boxplots indicate median, first and third quartiles of *n* = 8–10 samples; FC = fold change relative to control. Dots show outliers beyond 1.5-times the interquartile range, which is represented by whiskers. Kruskal–Wallis tests were separately performed for 6 h and 24 h samples. In case of a significant Kruskal–Wallis test result (*P < 0.05, **P < 0.01, ***P < 0.001), the Wilcoxon rank-sum test with Benjamini–Hochberg correction was used to test for differences between treatments. **a**–**f** In the event of significant results from Kruskal–Wallis test or ANOVA, significant differences between treatments as determined by the aforementioned post-hoc tests (*P* < 0.05) are indicated by different capital letters (6 h samples) and lowercase letters (24 h samples)

Levels of SA were significantly higher in locally treated EF leaves than F leaves after a 24 h feeding period. This egg-mediated effect on the SA concentration in feeding-damaged elm leaves was not detectable after a 6 h feeding period. Neither egg treatment nor feeding damage per se affected SA levels, regardless of the time point of sampling. No treatment effects on SA levels were detected in systemic leaves (Fig. 2a; Tables S4, S5).

Levels of JA and JA-Ile were, as expected, significantly higher in both locally treated F and EF leaves. This feeding-induced effect was detectable after both a 6 h and 24 h larval feeding period (Fig. 2b, c). Prior egg deposition did not enhance the feeding-induced JA levels in locally treated leaves. In systemic F and EF leaves, concentrations of JA and JA-Ile increased in response to feeding but remained below 5 ng g^−1^ fresh weight in all treatments (Tables S4, S5).

Levels of ABA changed neither in response to the egg treatment, to larval feeding, nor to a combination of both treatments (Tables S4, S5). This lack of treatment effects was observed in local and systemic leaves both 6 h and 24 h after the onset of feeding damage.

In summary, concentrations of SA were only enhanced in local, previously egg-treated leaves after 24 h of feeding, while larval feeding increased JA and JA-Ile concentrations after both 6 h and 24 h of feeding in locally treated leaves.

### Effects of egg deposition and larval feeding on elm genes involved in the phenylpropanoid pathway

The effects of egg deposition on expression of genes involved in the phenylpropanoid pathway were detectable only for *PAL.* Prior elm leaf beetle egg deposition affected transcript levels of *PAL* in locally treated elm leaves exposed to 24 h of larval feeding. These EF leaves showed significantly higher transcript levels than F leaves (Fig. 2d). This egg-mediated enhancer effect on *PAL* expression in local, feeding-induced elm leaves was not detectable after a 6 h feeding period (Fig. 2d). In the absence of larval feeding damage, *PAL* expression levels were not enhanced in locally treated E leaves at the 6 h or 24 h leaf sampling time points. In local leaves, whether E or EF leaves, expression of none of the other tested candidate genes—*4CL*, *HCT*, *CAD*, *COMT*, *FLS/F3H*, *F3′H*, *ANS*—was affected by elm leaf beetle egg deposition (Table S6). When considering gene expression in systemic E and EF leaves after a 24 h feeding period, no effects of prior egg deposition were detected on transcript levels of the genes tested (Table S7).

Larval feeding induced expression of *PAL*, *HCT* and *ANS* in locally treated leaves with and without prior egg deposition*.* While *PAL* and *ANS* showed enhanced transcript levels both after 6 h and 24 h of larval feeding in locally treated leaves, *HCT* expression was significantly higher only after a 6 h feeding period, and only when compared to egg-treated E leaves without feeding damage (Fig. 2d–f). In local leaves, expression of none of the other tested candidate genes—*4CL*, *HCT*, *CAD*, *COMT*, *FLS/F3H*, *F3′H*—was affected by 24 h of larval feeding (Table S6). When considering systemic effects of larval feeding, *PAL* and *ANS* transcript levels were significantly higher after a 24 h larval feeding period, regardless of whether the leaves received an egg treatment beforehand (Table S7). Larval feeding for 24 h did not affect expression levels of any of the other tested candidate genes in systemic leaves (Table S7).

As such, insect egg deposition amplifies the feeding-induced expression of a homologue to *phenylalanine ammonia lyase* (*PAL*), a gene encoding an enzyme at the entrance of the phenylpropanoid pathway, in elm leaves. This egg-mediated enhancer effect was only detectable in locally treated leaves after a 24 h larval feeding period.

### Effects of egg deposition and larval feeding on metabolites of the phenylpropanoid pathway

**Fig. 3:**
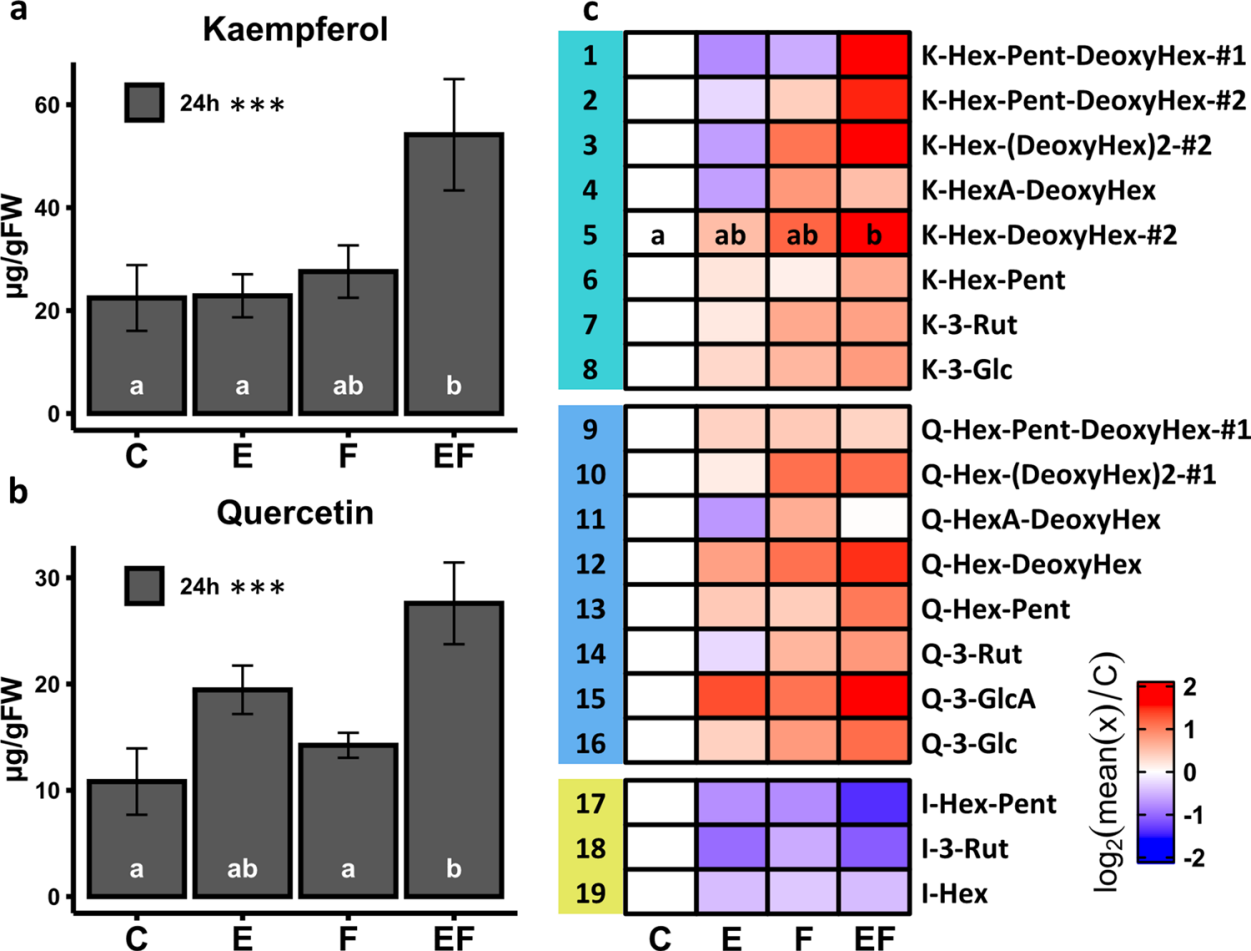
Impact of elm leaf beetle egg deposition and larval feeding on *Ulmus minor* flavonoid content. Total concentrations of kaempferol (**a**) and quercetin (**b**) as analysed by HPLC–DAD of acid-hydrolysed methanolic leaf extracts. Heatmap in **c** shows log_2_ fold change of metabolite levels of flavonol glycosides detected in methanolic leaf extracts by UHPLC/ESI–QTOFMS. Relative peak areas were standardised to leaf fresh weight. Log_2_ fold change relative to control was calculated by log_2_ of the ratio of the mean peak area per metabolite in a treated leaf relative to the mean of the respective metabolite peak area in the control*.* Numbers in **c** refer to compound numbers mentioned in the text; kaempferol (1–8), quercetin (9–16) and isorhamnetin derivatives (17–19). C = untreated control leaves, E = locally egg-treated leaves, F = locally feeding-damaged leaves, 24 h feeding period, EF = locally egg-treated, feeding-damaged leaves, 24 h feeding period. Statistics in **a**–**c** ANOVA, in case of significance (*P* < 0.05) followed by Tukey test as post-hoc test with single-step adjusted *P* values. In **a** and **b** ***ANOVA *P* < 0.001. In **a**–**c** different letters indicate significant differences between treatments (Tukey test *P* < 0.05); *n* = 9–10

HPLC–DAD analysis of flavonol aglycones in locally treated leaves revealed that levels of kaempferol and quercetin were significantly higher in 24 h feeding-damaged elm leaves with prior egg deposition than in untreated control leaves (Fig. 3a, b; Table S8). Neither egg deposition nor feeding damage alone induced a significant increase in concentrations of these compounds. The quercetin concentrations of F and EF leaves differed significantly. The kaempferol concentration was almost doubled in EF leaves compared to F leaves; this difference was marginally significant (*P* = 0.06).

Using UHPLC/ESI–QTOFMS, we detected a total of 19 flavonol glycosides in methanolic extracts of locally treated elm leaves. We quantified eight kaempferol derivatives, eight quercetin derivatives and three isorhamnetin derivatives (Fig. 3c; Tables S3, S9). Overall, the variability of biological samples was relatively high. We recorded a significant increase in concentration of only one kaempferol glycoside when comparing EF leaves to untreated controls (Fig. 3c; compound #5). The concentrations of compounds #1–3 showed a similar pattern across the differently treated leaves, with the highest concentrations in EF leaves. Concentrations of five quercetin derivatives showed a similar pattern across treatments to that of the quercetin aglycone (compare Fig. 3b, c), with the highest concentrations in EF treated leaves (Fig. 3c; compounds #12–16). Compounds #4 and 11 were the only compounds with lower concentrations in EF leaves than in F leaves. Concentrations of all identified isorhamnetin derivatives slightly decreased in E, F and EF leaves as compared to untreated controls (Fig. 3c; compounds #17–19).

**Fig. 4:**
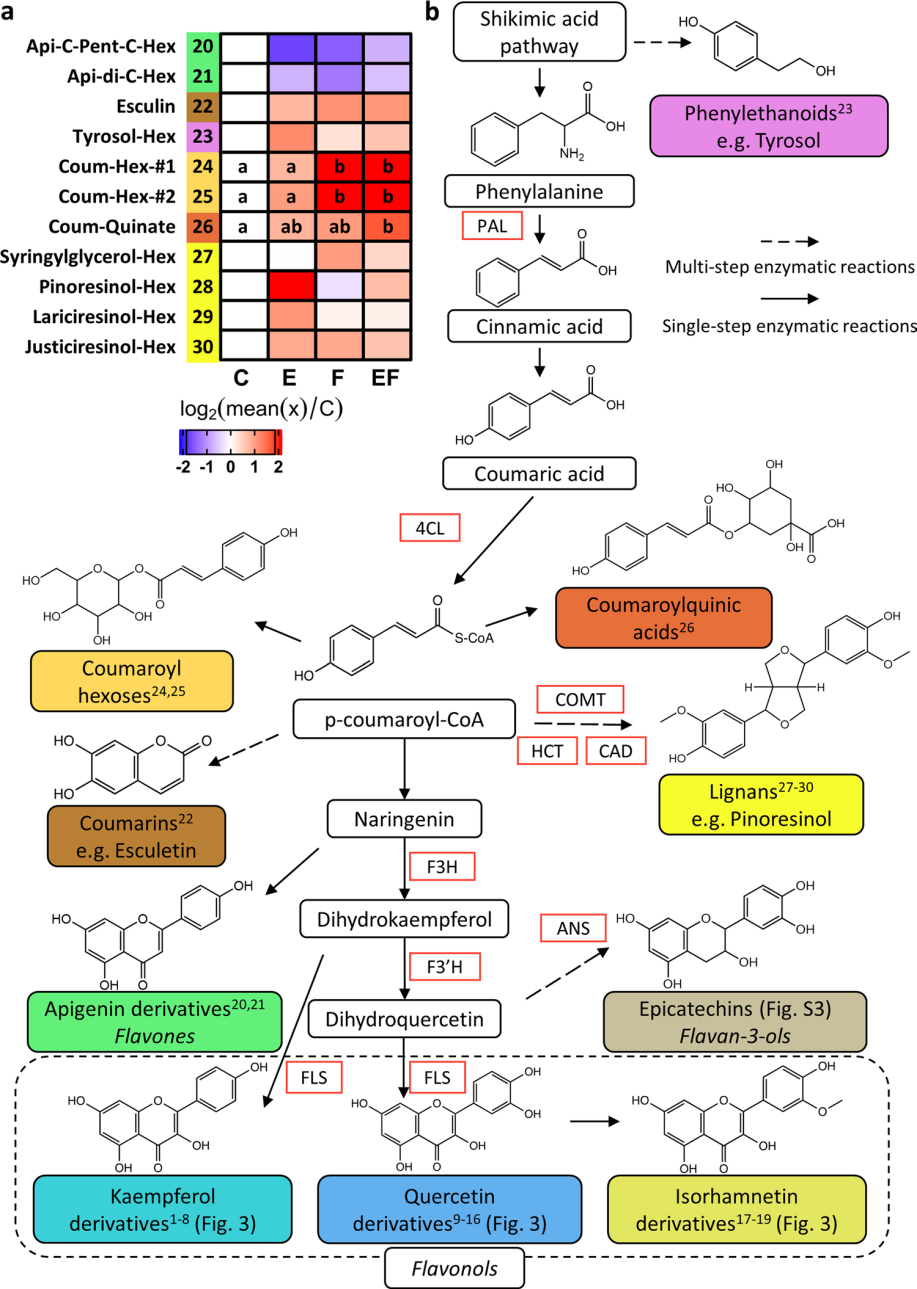
Impact of elm leaf beetle egg deposition and larval feeding on putatively identified *Ulmus minor* phenylpropanoid metabolites. UHPLC/ESI–QTOFMS-analysis of semipolar compounds present in a methanolic elm leaf extract. **a** Heatmap shows log_2_ fold change of metabolite levels in a treated leaf relative to an untreated control leaf. Relative peak areas were standardised to leaf fresh weight. Log_2_ fold change relative to control was calculated by log_2_ of the ratio of the mean peak area per metabolite in a treated leaf relative to the mean of the peak area of the respective metabolite in the control*.* Colouring and numbers next to substance names code for pathway affiliation (see **b**). Treated leaves are labelled as follows: E = locally egg-treated leaves; F = locally feeding-damaged leaves, 24 h feeding period; EF = locally egg-treated, feeding-damaged leaves, 24 h feeding period; C = untreated control leaves. Statistics: ANOVA, in case of significance (*P* < 0.05) followed by Tukey test as post-hoc test with single-step adjusted *P* values. Different letters indicate significant differences between treatments (*P* < 0.05); *n* = 9–10. Compounds referred to twice are isomeric structures with the same molecular weight but differing retention times. **b** Simplified visualisation of the phenylpropanoid pathway. Colours and indexed numbers represent the compound affiliation of respective compounds in the heatmap shown in a. Enzymes catalysing selected biosynthetic steps are indicated; PAL phenylalanine ammonia lyase, 4CL 4-coumarate ligase, HCT shikimate *O*-hydroxycinnamoyl-transferase, CAD cinnamyl alcohol dehydrogenase, COMT caffeic acid 3-*O*-methyltransferase, F3H flavanone 3-hydroxylase, F3′H flavonoid 3′-monooxygenase, FLS flavonol synthase, ANS leucoanthocyanidin dioxygenase

To elucidate how egg deposition and larval feeding might affect phenylpropanoid compounds other than the flavonol glycosides, we subjected the LC/MS profiles of methanolic elm leaf extracts to a non-targeted analysis. We were able to detect 124 features, which differed in their peak area in at least one of the pairwise comparisons of untreated controls with E, F and EF leaves and of the comparison between F and EF leaves. Further analysis of these features allowed us to assign them to 20 compounds, among which we could tentatively identify 12 metabolites. Of these 12, 11 could be further specified as shikimate/phenylpropanoid derivatives (compounds #20–30; Fig. 4a, b; Tables S3, S10), and the other as suberic acid (compound #31, Table S3). No reliable compound classification was possible for the remaining eight metabolites (compounds #32–39, Table S3). Coumaroyl-quinate (compound #26) was the only tentatively identified phenylpropanoid compound to appear at a significantly increased concentration in EF leaves compared to C leaves. Levels of two isomeric coumaroyl-hexoses were increased by feeding in both F and EF leaves (compounds #24, 25). Similar to isorhamnetin derivatives, levels of two apigenin derivatives decreased slightly, but not significantly, in response to all treatments when compared to controls (compounds #20, 21).

In addition to the flavonol glycosides shown in Fig. 3, we detected the flavan-3-ols catechin, epicatechin and their dimers and trimers in the methanolic elm leaf extract. Of the seven substances identified, only epicatechin showed a tentative increase in egg-treated E leaves and a tentative decrease in EF leaves as compared to untreated controls; however, none of the comparisons revealed any significant differences (Fig. S3; Tables S3, S9).

Taken together, the metabolite analyses showed that feeding damage by neonate elm leaf beetle larvae for 24 h upon egg-free leaves did not result in enhanced concentrations of phenylpropanoid derivatives, except for coumaric acid glycosides. Likewise, egg deposition per se did not lead to significant changes in phenylpropanoid concentrations. However, elm leaves with prior egg deposition accumulated higher concentrations of the flavonoids kaempferol and quercetin in response to larval feeding damage. These two flavonols were detected in several glycosylated forms.

## Discussion

Our study shows that insect egg deposition on a deciduous tree significantly shapes that tree’s phytohormonal and metabolite response to larvae hatching from the eggs. The effects of elm leaf beetle egg deposition on feeding-damaged elm leaves are manifested in enhanced levels of the phytohormone SA and of some phenylpropanoid derivatives after 24 h feeding by neonate larvae. Expression levels of *PAL*, which encodes the “gateway” enzyme (Zhang and Liu 2015) at the entrance of the phenylpropanoid pathway, were increased after feeding in previously egg-laden leaves to a greater extent than in egg-free, feeding-damaged leaves. However, in feeding-damaged leaves, expression levels of other genes involved in phenylpropanoid biosynthesis were not affected by prior egg deposition.

### Effects of egg deposition and larval feeding on elm phytohormone levels

In elm, the induction of JA and its bioactive form, JA-Ile, by larval feeding for 6 h and 24 h was independent of prior egg deposition. In another perennial plant species, the bittersweet nightshade *Solanum dulcamara*, increases of JA and JA-Ile levels after a 24 h larval feeding period were also independent of prior egg deposition (Geuss et al. 2018). In contrast, JA and JA-Ile levels in other species with shorter longevity were affected by prior egg deposition. However, concentrations of these phytohormones were measured very early after the onset of damage. For instance, higher JA levels were detected in egg-treated tomato plants 30 min and 60 min after wounding (Kim et al. 2012). In *Arabodpsis thaliana*, Valsamakis et al. (2020) found enhanced JA-Ile levels in egg-treated leaves after a 3 h larval feeding period when compared to egg-free, feeding-damaged leaves, but not after a 12 h feeding period. Future studies will need to investigate whether previously egg-laden elm leaves show enhanced JA levels very early on after the onset of larval feeding.

Levels of SA were significantly enhanced in previously egg-treated elm leaves after a larval feeding period of 24 h. Similarly, significantly enhanced SA levels were detected in *A. thaliana* when laden with eggs by the butterfly *Pieris brassicae* and subsequently damaged by its hatching larvae for 24 h (Lortzing et al. 2019; Valsamakis et al. 2020). In contrast, moth egg depositions upon the annual species *Nicotiana attenuata* and the perennial *S. dulcamara* did not affect SA levels in subsequently larval feeding-damaged leaves (Drok et al. 2018). Thus, the SA response of plants to egg deposition and subsequent larval feeding damage varies according to the interacting plant and insect species. In the species studied to date, there is no apparent relationship between plant longevity and the increased SA content in egg-laden, feeding-damaged leaves.

Enhanced SA levels in egg-treated, feeding-damaged elm leaves are accompanied by enhanced transcript levels of *PAL.* Enhanced activity of PAL might contribute to the higher levels of SA in EF leaves, since this phytohormone might be produced via a PAL-dependent pathway in elm. While numerous studies indicate that SA in *A. thaliana* is predominantly produced via the isochorismate (IC) pathway (Dempsey et al. 2011; Rekhter et al. 2019), the PAL-dependent pathway is an additional biosynthetic route to SA. Along this PAL-dependent pathway, phenylalanine is converted into *trans*-cinnamic acid, which may then be further processed via different steps to *ortho*-coumaric acid or benzoic acid as immediate precursors of SA. It is unknown which pathway is (primarily) used by elm for biosynthesis of SA. If SA is generated in elm primarily via the PAL pathway, it would seem to be less sensitive to egg deposition alone than the IC pathway, since neither *PAL* expression nor SA levels in elm change in response to egg deposition per se*.* In *A. thaliana*, SA levels are known to increase in response to *P. brassicae* egg deposition (Hilfiker et al. 2014; Bonnet et al. 2017; Gouhier-Darimont et al. 2019; Lortzing et al. 2019). Furthermore, *A. thaliana SID2*, which encodes isochorismate synthase 1, was found to be significantly induced by insect egg deposition alone (Valsamakis et al. 2020), thus suggesting a sensitivity in the IC pathway to insect eggs.

An increase in SA levels is usually thought to antagonise JA-regulated plant defensive responses (e.g., Koornneef and Pieterse 2008; Thaler et al. 2012). However, Austel et al. (2016) showed that elm defences against elm leaf beetle larvae were more efficient when leaves had received the beetle’s eggs prior to larval feeding damage. There is increasing evidence that the relationship between SA and JA is more complex than simple antagonism, with neutral and coordinated effects occurring too, often depending on the magnitude and timing of induction (Schenk et al. 2000; Mur et al. 2006; Salas-Marina et al. 2011; Rostás et al. 2013; Liu et al. 2016).

### Effects of egg deposition and larval feeding on elm genes involved in the phenylpropanoid pathway

PAL is required for the production of phenylalanine-derived compounds, which are involved in anti-herbivore defences in numerous plant species (War et al. 2018). Several abiotic and biotic cues, including wounding and insect feeding damage, are well known for inducing upregulation of *PAL* gene expression and enzyme activity (Hartley and Firn 1989; Major and Constabel 2006; Ralph et al. 2006; Dreischhoff et al. 2020)*. PAL* is also known to be induced 3 days after insect egg deposition upon *A. thaliana* (Little et al. 2007). Furthermore, expression of *PAL* is inducible in elm by fungi causing Dutch elm disease (Martín et al. 2019 and references therein). *PAL* expression is not only inducible, but also primable, in wounded or phytopathogen-infected leaves by a pre-treatment with SA or with benzothiadiazole (BTH), a synthetic SA analogue (Kohler et al. 2002; Conrath et al. 2006, and references therein). However, until the present study, the primability of *PAL* expression in feeding-induced leaves due to an infestation-indicating cue, i.e., insect egg deposition, has not been investigated.

While feeding-induced *PAL* expression was enhanced by prior egg deposition upon elm leaves, no such egg-mediated effects were detected for the expression of other genes of the phenylpropanoid pathway. Larval feeding induced expression of elm sequences homologous to leucoanthocyanidin dioxygenase (*ANS*) and shikimate *O*-hydroxycinnamoyl-transferase (*HCT*) independent of prior egg deposition in both EF and F leaves. In contrast, expression of a *HCT* homologue in the bittersweet nightshade *S. dulcamara* was even downregulated in egg-free leaves damaged by *Spodoptera exigua* larval feeding, while transcript levels in previously egg-laden, feeding-damaged leaves were upregulated (Geuss et al. 2018); these findings indicate that *HCT* responses to feeding damage and egg deposition are specific to the plant–insect interaction in question.

None of the other genes studied here were expressed differently in response to larval feeding and/or egg deposition. That larval feeding did not induce these genes is surprising, since several genes downstream of *PAL* in the phenylpropanoid pathway are known to be inducible by wounding or insect feeding (e.g., *4CL*: Soltani et al. 2006; *CAD:* Barakat et al. 2010; *F3′H:* Onkokesung et al. 2014*)*. It could be that the damage inflicted by neonate larvae feeding for 24 h upon the elm leaves was too minimal to induce transcription of these genes. Another possibility is that we might have analysed isoforms of these genes which are not inducible by wounding; for example, two isoforms of *4CL* in *A. thaliana* have been shown to be wound-inducible, but a third one did not respond to wounding (Ehlting et al. 1999).

### Effects of egg deposition and larval feeding on elm flavonoid levels

Neither egg deposition nor feeding damage alone changed levels of elm leaf flavonoids. However, the flavonol core structures, kaempferol and quercetin, were produced in significantly higher concentrations in egg-treated, feeding-damaged elm leaves than in untreated controls. Similarly, total levels of quercetin and kaempferol derivatives were significantly higher in egg-treated, feeding-damaged *A. thaliana* leaves when compared to untreated controls and to egg-free, feeding-damaged leaves (Lortzing et al. 2019). Egg-treated, feeding-damaged tobacco plants (*N. attenuata*) have also shown significantly higher levels of a certain phenylpropanoid, caffeoylputrescine, than egg-free, damaged leaves, while egg deposition alone did not induce this phenylpropanoid (Bandoly et al. 2015). Hence, while the response of plants to insect egg deposition alone varies with the specific plant and insect species, in all interactions studied so far, the concentrations of phenylpropanoids were higher when egg deposition preceded larval feeding.

The changes of elm metabolite concentrations in response to the study treatments hardly matched the changes in expression of genes involved in their biosynthesis. We expected the enhanced levels of kaempferol and quercetin in EF leaves to be accompanied by enhanced transcript levels of *FLS/F3H* and *F3′H,* the genes involved in biosynthesis of these compounds. However, this was not the case. Likewise, Schulz et al. (2015) and Pastore et al. (2017) discovered only weak correlations between temperature-induced transcript and metabolite levels of flavonoids. We suggest that in elm increased levels of basic precursors of flavonol biosynthesis are provided by the egg-mediated, feeding-induced potentiation of *PAL* expression. However, since biosynthesis of phenylpropanoids is not exclusively regulated transcriptionally (Deng and Lu 2017; Yu et al. 2019; Sharma et al. 2019; Nabavi et al. 2020), it could be that post-transcriptional or post-translational mechanisms affected the production of kaempferol and quercetin in elm EF leaves. A previous RNA-seq analysis showed earlier and/or faster transcriptional regulation in elm EF than in F leaves after the onset of larval feeding; interestingly, a gene ontology term enrichment analysis indicated that among these early responding transcripts in EF leaves is a set of transcripts with a function in post-translational protein modification (Altmann et al. 2018). Future studies will need to investigate whether post-translational processes regulate flavonoid biogenesis in response to elm leaf beetle infestation.

Although it has been shown that a high concentration of a kaempferol glycoside causes increased mortality of elm leaf beetle larvae (Austel et al. 2016), the mode of action of flavonols on elm leaf beetle larvae is unknown. An early elm response at the onset of feeding by neonate larvae may be an efficient defence trait because young larvae may be especially sensitive to defensive phytochemicals (Zalucki et al. 2002).

## Concluding remarks

Our study has demonstrated that a tree species, *U. minor*, responds to the combination of insect egg deposition and feeding by enhancing *PAL* transcript levels, concentrations of SA, and the flavonols kaempferol and quercetin. When comparing elm responses to insect eggs and larval feeding with those of other plant species, no response pattern typical for perennial versus annual plant species was found. However, from an ecological perspective, the plant species studied here and elsewhere show similarly improved defences against larval feeding damage after having received egg depositions.

Differences between plant species’ phytohormonal, transcriptional and metabolic responses to insect eggs and larval feeding may not only be due to species-specific sensitivity to insect eggs, but also to species-specific kinetics of responses, meaning the time points used for taking measurements are crucial. Using the elm leaf sampling time points here, we did not detect effects of egg deposition alone on phytohormone levels, gene expression or metabolite levels. However, a previous study by Altmann et al. (2018) demonstrated that a few hundred elm genes show moderate differential expression very early on, i.e., 1 h after egg deposition; this early response to eggs was short-lived and later returned to control levels. Our metabolite study here indicates that this very early differential gene expression in response to elm leaf beetle eggs does not result in sustainable accumulation of phenylpropanoids or enhanced levels of phytohormones, which are maintained until later time points. Further studies need to address the question of *how* the elm’s response to eggs can potentiate the response to larval feeding damage, and in doing so will need to consider the impact of, for instance, egg-mediated epigenetic and chromatin-based modifications or small RNAs, which are known to regulate the priming of responses to stress in annual plant species (Rasmann et al. 2012; Lämke and Bäurle 2017; Hilker and Schmülling 2019).

### *Author contribution statement*

JS and BF performed and evaluated all experiments and analyses other than the LC–MS analyses, which were conducted by CB and evaluated by JS, BF and CB. MH conceptualised, designed and organised the study. JS and BF wrote a first draft of the manuscript, MH significantly contributed to a later draft, and all authors contributed to, and agreed upon, the final version.

## Supplementary Information

Below is the link to the electronic supplementary material.

Supplementary file1 (PDF 523 kb)

Supplementary file2 (XLSX 21 kb)

## Data Availability

The data supporting the findings of this study are available in the main text and online, in the Supplementary Data.

## Abbreviations

4CL: 4-Coumarate ligase
ANS: Leucoanthocyanidin dioxygenase
C: Untreated control
CAD: Cinnamyl alcohol dehydrogenase
COMT: Caffeic acid 3-*O*-methyltransferase
E: Standardised egg deposition treatment
EF: Standardised egg deposition plus larval feeding treatment
F: Larval feeding treatment
F3′H: Flavonoid 3′-monooxygenase
FLS/F3H: Flavonol synthase/flavanone 3-hydroxylase
HCT: Shikimate *O*-hydroxycinnamoyl-transferase
JA: Jasmonic acid
JA-Ile: Jasmonic acid-isoleucine
PAL: Phenylalanine ammonia lyase
SA: Salicylic acid

## References

[CR1] Altmann S, Muino JM, Lortzing V, Brandt R, Himmelbach A, Altschmied L, Hilker M (2018). Transcriptomic basis for reinforcement of elm antiherbivore defence mediated by insect egg deposition. Mol Ecol.

[CR2] Austel N, Eilers EJ, Meiners T, Hilker M (2016). Elm leaves ‘warned’ by insect egg deposition reduce survival of hatching larvae by a shift in their quantitative leaf metabolite pattern. Plant Cell Environ.

[CR3] Bandoly M, Hilker M, Steppuhn A (2015). Oviposition by *Spodoptera exigua* on *Nicotiana attenuata* primes induced plant defence against larval herbivory. Plant J.

[CR4] Barakat A, Bagniewska-Zadworna A, Frost CJ, Carlson JE (2010). Phylogeny and expression profiling of *CAD and CAD-like* genes in hybrid *Populus* (*P. deltoides* × *P. nigra*): evidence from herbivore damage for subfunctionalization and functional divergence. BMC Plant Biol.

[CR5] Bate-Smith EC, Richens RH (1973). Flavonoid chemistry and taxonomy in *Ulmus*. Biochem Syst Ecol.

[CR6] Beyaert I, Köpke D, Stiller J, Hammerbacher A, Yoneya K, Schmidt A, Gershenzon J, Hilker M (2012). Can insect egg deposition ‘warn’ a plant of future feeding damage by herbivorous larvae?. Proc Biol Sci.

[CR7] Boeckler GA, Gershenzon J, Unsicker SB (2011). Phenolic glycosides of the Salicaceae and their role as anti-herbivore defenses. Phytochemistry.

[CR8] Bonnet C, Lassueur S, Ponzio C, Gols R, Dicke M, Reymond P (2017). Combined biotic stresses trigger similar transcriptomic responses but contrasting resistance against a chewing herbivore in *Brassica nigra*. BMC Plant Biol.

[CR9] Boyd IL, Freer-Smith PH, Gilligan CA, Godfray HCJ (2013). The consequence of tree pests and diseases for ecosystem services. Science.

[CR10] Büchel K, Fenning T, Gershenzon J, Hilker M, Meiners T (2016). Elm defence against herbivores and pathogens: morphological, chemical and molecular regulation aspects. Phytochem Rev.

[CR11] Caldwell E, Read J, Sanson GD (2016). Which leaf mechanical traits correlate with insect herbivory among feeding guilds?. Ann Bot.

[CR12] Conrath U, Beckers GJM, Flors V (2006). Priming: getting ready for battle. Mol Plant Microbe Interact.

[CR13] Conrath U, Beckers GJM, Langenbach CJG, Jaskiewicz MR (2015). Priming for enhanced defence. Annu Rev Phytopathol.

[CR14] Dempsey DA, Vlot AC, Wildermuth MC, Klessig DF (2011). Salicylic acid biosynthesis and metabolism. Arabidopsis Book.

[CR15] Deng Y, Lu S (2017). Biosynthesis and regulation of phenylpropanoids in plants. Crit Rev Plant Sci.

[CR16] Dreischhoff S, Das IS, Jakobi M, Kasper K, Polle A (2020). Local responses and systemic induced resistance mediated by ectomycorrhizal fungi. Front Plant Sci.

[CR17] Drok S, Bandoly M, Stelzer S, Lortzing T, Steppuhn A (2018). Moth oviposition shapes the species-specific transcriptional and phytohormonal response of *Nicotiana attenuata* to larval feeding. Sci Rep.

[CR18] Ehlting J, Büttner D, Wang Q, Douglas CJ, Somssich IE, Kombrink E (1999). Three 4-coumarate:coenzyme A ligases in *Arabidopsis thaliana* represent two evolutionarily divergent classes in angiosperms. Plant J.

[CR19] Farmer EE, Gao Y-Q, Lenzoni G, Wolfender J-L, Wu Q (2020). Wound- and mechanostimulated electrical signals control hormone responses. New Phytol.

[CR20] Fox J, Weisberg S (2018). An R companion to applied regression.

[CR21] Frost CJ, Mescher MC, Carlson JE, Moraes CMD (2008). Plant defense priming against herbivores: getting ready for a different battle. Plant Physiol.

[CR22] Geuss D, Lortzing T, Schwachtje J, Kopka J, Steppuhn A (2018). Oviposition by *Spodoptera exigua* on *Solanum dulcamara* alters the plant’s response to herbivory and impairs larval performance. Int J Mol Sci.

[CR23] Gouhier-Darimont C, Stahl E, Glauser G, Reymond P (2019). The *Arabidopsis* lectin receptor kinase LecRK-I.8 is involved in insect egg perception. Front Plant Sci.

[CR24] Gu Z, Eils R, Schlesner M (2016). Complex heatmaps reveal patterns and correlations in multidimensional genomic data. Bioinformatics.

[CR25] Hartley SE, Firn RD (1989). Phenolic biosynthesis, leaf damage, and insect herbivory in birch (*Betula pendula*). J Chem Ecol.

[CR26] Haukioja E (1991). Induction of defenses in trees. Annu Rev Entomol.

[CR27] Hertog MGL, Hollman PCH, Katan MB (1992). Content of potentially anticarcinogenic flavonoids of 28 vegetables and 9 fruits commonly consumed in the Netherlands. J Agric Food Chem.

[CR28] Hilfiker O, Groux R, Bruessow F, Kiefer K, Zeier J, Reymond P (2014). Insect eggs induce a systemic acquired resistance in Arabidopsis. Plant J.

[CR29] Hilker M, Fatouros NE (2016). Resisting the onset of herbivore attack: plants perceive and respond to insect eggs. Curr Opin Plant Biol.

[CR30] Hilker M, Schmülling T (2019). Stress priming, memory, and signalling in plants. Plant Cell Environ.

[CR31] Hilker M, Schwachtje J, Baier M (2016). Priming and memory of stress responses in organisms lacking a nervous system. Biol Rev.

[CR32] Hothorn T, Bretz F, Westfall P (2008). Simultaneous inference in general parametric models. Biom J.

[CR33] Ikoma Y, Yano M, Ogawa K, Yoshioka T, Xu ZC, Hisada S, Omura M, Moriguchi T (1996). Isolation and evaluation of RNA from polysaccharide-rich tissues in fruit for quality by cDNA library construction and RT-PCR. J Jpn Soc Hortic Sci.

[CR34] Karban R (2011). The ecology and evolution of induced resistance against herbivores. Funct Ecol.

[CR35] Kim J, Tooker JF, Luthe DS, Moraes CMD, Felton GW (2012). Insect eggs can enhance wound response in plants: a study system of tomato *Solanum lycopersicum* L. and *Helicoverpa zea* Boddie. PLoS One.

[CR36] Kohler A, Schwindling S, Conrath U (2002). Benzothiadiazole-induced priming for potentiated responses to pathogen infection, wounding, and infiltration of water into leaves requires the *NPR1*/*NIM1* gene in Arabidopsis. Plant Physiol.

[CR37] Koornneef A, Pieterse CMJ (2008). Cross talk in defense signaling. Plant Physiol.

[CR38] Lämke J, Bäurle I (2017). Epigenetic and chromatin-based mechanisms in environmental stress adaptation and stress memory in plants. Genome Biol.

[CR39] Li T, Blande JD (2017). Volatile-mediated within-plant signaling in hybrid aspen: required for systemic responses. J Chem Ecol.

[CR40] Little D, Gouhier-Darimont C, Bruessow F, Reymond P (2007). Oviposition by pierid butterflies triggers defense responses in Arabidopsis. Plant Physiol.

[CR41] Liu L, Sonbol F-M, Huot B, Gu Y, Withers J, Mwimba M, Yao J, He SY, Dong X (2016). Salicylic acid receptors activate jasmonic acid signalling through a non-canonical pathway to promote effector-triggered immunity. Nat Commun.

[CR42] Livak KJ, Schmittgen TD (2001). Analysis of relative gene expression data using real-time quantitative PCR and the 2^−ΔΔCT^ method. Methods.

[CR43] Lortzing V, Oberländer J, Lortzing T, Tohge T, Steppuhn A, Kunze R, Hilker M (2019). Insect egg deposition renders plant defence against hatching larvae more effective in a salicylic acid-dependent manner. Plant Cell Environ.

[CR44] Major IT, Constabel CP (2006). Molecular analysis of poplar defense against herbivory: comparison of wound- and insect elicitor-induced gene expression. New Phytol.

[CR45] Martín JA, Witzell J, Blumenstein K, Rozpedowska E, Helander M, Sieber TN, Gil L (2013). Resistance to Dutch elm disease reduces presence of xylem endophytic fungi in elms (*Ulmus* spp.). PLoS One.

[CR46] Martín JA, Sobrina-Plata J, Rodriguez-Calcerrada J, Collada C, Gil L (2019). Breeding and scientific advances in the fight against Dutch elm disease: will they allow the use of elms in forest restoration?. New For.

[CR47] Mattila P, Astola J, Kumpulainen J (2000). Determination of flavonoids in plant material by HPLC with diode-array and electro-array detections. J Agric Food Chem.

[CR48] Mauch-Mani B, Baccelli I, Luna E, Flors V (2017). Defense priming: an adaptive part of induced resistance. Annu Rev Plant Biol.

[CR49] Meiners T, Hilker M (2000). Induction of plant synomones by oviposition of a phytophagous insect. J Chem Ecol.

[CR50] Mur LAJ, Kenton P, Atzorn R, Miersch O, Wasternack C (2006). The outcomes of concentration-specific interactions between salicylate and jasmonate signaling include synergy, antagonism, and oxidative stress leading to cell death. Plant Physiol.

[CR51] Nabavi SM, Šamec D, Tomczyk M (2020). Flavonoid biosynthetic pathways in plants: versatile targets for metabolic engineering. Biotechnol Adv.

[CR52] Onkokesung N, Reichelt M, van Doorn A, Schuurink RC, van Loon JJA, Dicke M (2014). Modulation of flavonoid metabolites in *Arabidopsis thaliana* through overexpression of the MYB75 transcription factor: role of kaempferol-3,7-dirhamnoside in resistance to the specialist insect herbivore *Pieris brassicae*. J Exp Bot.

[CR53] Pastore C, Dal Santo S, Zenoni S, Movahed N, Allegro G, Valentini G, Filippetti I, Tornielli GB (2017). Whole plant temperature manipulation affects flavonoid metabolism and the transcriptome of grapevine berries. Front Plant Sci.

[CR54] Perdiguero P, Venturas M, Cervera MT, Gil L, Collada C (2015). Massive sequencing of *Ulmus minor*’s transcriptome provides new molecular tools for a genus under the constant threat of Dutch elm disease. Front Plant Sci.

[CR55] R Core Team (2020) R: a language and environment for statistical computing. R Foundation for Statistical Computing, Vienna

[CR56] Ralph S, Oddy C, Cooper D (2006). Genomics of hybrid poplar (*Populus trichocarpa× deltoides*) interacting with forest tent caterpillars (*Malacosoma disstria*): normalized and full-length cDNA libraries, expressed sequence tags, and a cDNA microarray for the study of insect-induced defences in poplar. Mol Ecol.

[CR57] Rasmann S, Vos MD, Casteel CL, Tian D, Halitschke R, Sun JY, Agrawal AA, Felton GW, Jander G (2012). Herbivory in the previous generation primes plants for enhanced insect resistance. Plant Physiol.

[CR58] Rekhter D, Lüdke D, Ding Y, Feussner K, Zienkiewicz K, Lipka V, Wiermer M, Zhang Y, Feussner I (2019). Isochorismate-derived biosynthesis of the plant stress hormone salicylic acid. Science.

[CR59] Reymond P (2013). Perception, signaling and molecular basis of oviposition-mediated plant responses. Planta.

[CR60] Rostás M, Winter TR, Borkowski L, Zeier J (2013). Copper and herbivory lead to priming and synergism in phytohormones and plant volatiles in the absence of salicylate-jasmonate antagonism. Plant Signal Behav.

[CR61] Salas-Marina MA, Silva-Flores MA, Uresti-Rivera EE, Castro-Longoria E, Herrera-Estrella A, Casas-Flores S (2011). Colonization of *Arabidopsis* roots by *Trichoderma atroviride* promotes growth and enhances systemic disease resistance through jasmonic acid/ethylene and salicylic acid pathways. Eur J Plant Pathol.

[CR62] Santamour FS (1972). Flavonoid distribution in *Ulmus*. Bull Torrey Bot Club.

[CR63] Schenk PM, Kazan K, Wilson I, Anderson JP, Richmond T, Somerville SC, Manners JM (2000). Coordinated plant defense responses in *Arabidopsis* revealed by microarray analysis. Proc Natl Acad Sci USA.

[CR64] Schulz E, Tohge T, Zuther E, Fernie AR, Hincha DK (2015). Natural variation in flavonol and anthocyanin metabolism during cold acclimation in *Arabidopsis thaliana* accessions. Plant Cell Environ.

[CR65] Sharma A, Shahzad B, Rehman A, Bhardwaj R, Landi M, Zheng B (2019). Response of phenylpropanoid pathway and the role of polyphenols in plants under abiotic stress. Molecules.

[CR66] Smith CA, Want EJ, O’Maille G, Abagyan R, Siuzdak G (2006). XCMS: processing mass spectrometry data for metabolite profiling using nonlinear peak alignment, matching, and identification. Anal Chem.

[CR67] Soltani BM, Ehlting J, Hamberger B, Douglas CJ (2006). Multiple cis-regulatory elements regulate distinct and complex patterns of developmental and wound-induced expression of *Arabidopsis thaliana* 4CL gene family members. Planta.

[CR68] Stam JM, Kroes A, Li Y, Gols R, van Loon JJA, Poelman EH, Dicke M (2014). Plant interactions with multiple insect herbivores: from community to genes. Annu Rev Plant Biol.

[CR69] Thaler JS, Humphrey PT, Whiteman NK (2012). Evolution of jasmonate and salicylate signal crosstalk. Trends Plant Sci.

[CR70] Tohge T, de Souza LP, Fernie AR (2017). Current understanding of the pathways of flavonoid biosynthesis in model and crop plants. J Exp Bot.

[CR71] Tscharntke T, Thiessen S, Dolch R, Boland W (2001). Herbivory, induced resistance, and interplant signal transfer in *Alnus glutinosa*. Biochem Syst Ecol.

[CR72] Turlings TCJ, Erb M (2018). Tritrophic interactions mediated by herbivore-induced plant volatiles: mechanisms, ecological relevance, and application potential. Annu Rev Entomol.

[CR73] Valsamakis G, Bittner N, Fatouros NE, Kunze R, Hilker M, Lortzing V (2020). Priming by timing: *Arabidopsis thaliana* adjusts its priming response to *Lepidoptera* eggs to the time of larval hatching. Front Plant Sci.

[CR74] Vandesompele J, De Preter K, Pattyn F, Poppe B, Van Roy N, De Paepe A, Speleman F (2002). Accurate normalization of real-time quantitative RT-PCR data by geometric averaging of multiple internal control genes. Genome Biol.

[CR75] Vogt T (2010). Phenylpropanoid biosynthesis. Mol Plant.

[CR84] Wang L, Halitschke R, Kang J-H, Berg A, Harnisch F, Baldwin IT (2007). Independently silencing two JAR family members impairs levels of trypsin proteinase inhibitors but not nicotine. Planta.

[CR76] War AR, Taggar GK, Hussain B, Taggar MS, Nair RM, Sharma HC (2018). Plant defence against herbivory and insect adaptations. AoB Plants.

[CR77] Wickham H (2011). The split-apply-combine strategy for data analysis. J Stat Softw.

[CR78] Wickham H (2016). ggplot2: Elegant graphics for data analysis.

[CR79] Wilkinson SW, Magerøy MH, López Sánchez A, Smith LM, Furci L, Cotton TEA, Krokene P, Ton J (2019). Surviving in a hostile world: plant strategies to resist pests and diseases. Annu Rev Phytopathol.

[CR80] Wu J, Baldwin IT (2010). New insights into plant responses to the attack from insect herbivores. Annu Rev Genet.

[CR81] Yu S, Kim H, Yun D-J, Suh MC, Lee B (2019). Post-translational and transcriptional regulation of phenylpropanoid biosynthesis pathway by Kelch repeat F-box protein SAGL1. Plant Mol Biol.

[CR82] Zalucki MP, Clarke AR, Malcolm SB (2002). Ecology and behavior of first instar larval Lepidoptera. Annu Rev Entomol.

[CR83] Zhang X, Liu C-J (2015). Multifaceted regulations of gateway enzyme phenylalanine ammonia-lyase in the biosynthesis of phenylpropanoids. Mol Plant.

