# Supplementary material for: Responses to larval herbivory in the phenylpropanoid pathway of Ulmus minor are boosted by prior insect egg deposition
Source: Planta. 2021 Dec 8;255(1):16. doi: 10.1007/s00425-021-03803-0 (PMC8654711; doi:10.1007/s00425-021-03803-0)
Supplement: Supplementary file 1 — Supplementary file1 (PDF 523 kb) [file 425_2021_3803_MOESM1_ESM.pdf]

SUPPLEMENTARY FILE 1

Journal: Planta

Article Title: Responses to larval herbivory in the phenylpropanoid pathway of *Ulmus minor* are boosted by prior insect egg deposition

Authors: Johanna Schott[1], Benjamin Fuchs[1, 3], Christoph Böttcher[2] and Monika Hilker[1]

[1] Department of Applied Zoology/Animal Ecology, Dahlem Centre of Plant Sciences, Freie Universität Berlin, Haderslebener Str. 9, 12163 Berlin, Germany

[2] Julius Kühn Institute (JKI) –Federal Research Centre for Cultivated Plants, Institute for Ecological Chemistry, Plant Analysis and Stored Product Protection, Königin-Luise-Str. 19, 14195 Berlin, Germany

[3] Current address: Biodiversity Unit, University of Turku, 20014 Turku, Finland

Authors with equal contribution: Johanna Schott, Benjamin Fuchs

Corresponding author: Monika Hilker, monika.hilker@fu-berlin.de, Tel. +49 30 838 55913

Supplementary Information: Overview

The following supplementary data are available for this article in this document, except for Table S3 (see separate Excel file):

Protocols

- Phytohormone extraction and measurement
- RNA extraction
- HPLC-DAD settings for analyses of flavonol aglycones
- UHPLC/ESI-QTOFMS settings for untargeted analyses and quality control
- Pre-processing of untargeted UHPLC/ESI-QTOFMS data of semipolar compounds
- Compound annotation by UHPLC/ESI-QTOFMS in targeted-MS/MS mode

Figures

- **Fig. S1** Detection of *O*-glycosylated flavonols via their protonated aglycone using all-ion fragmentation
- **Fig. S2** Detection of flavan-3-ols (catechins, epicatechins) and derived dimeric and trimeric proanthocyanidins via their deprotonated molecular ions
- **Fig. S3** Impact of elm leaf beetle egg deposition and larval feeding on *Ulmus minor* (epi-)catechin contents

Tables

- **Table S1** Primers for putative *Ulmus minor* genes involved in the biosynthesis of phenylpropanoids
- **Table S2** Reference genes and their primer sequences
- **Table S3** MS_MS analytical data (not included in this document; see separate supplemental Excel file)
- **Table S4** Phytohormone levels in locally treated *Ulmus minor* leaves after 24 h of larval feeding and at an equivalent time point for treatments without larval feeding
- **Table S5** Phytohormone levels in *Ulmus minor* leaves adjacent to treated leaves (‘systemic’ leaves) after 24 h of larval feeding and at an equivalent time point for treatments without larval feeding
- **Table S6** Expression of homologues of phenylpropanoid synthesis-related genes in local *Ulmus minor* leaves
- **Table S7** Expression of homologues of phenylpropanoid synthesis-related genes in systemic *Ulmus minor* leaves
- **Table S8** Concentrations of kaempferol and quercetin aglycones derived from acidified (hydrolysed) *Ulmus minor* extracts
- **Table S9** Relative quantification of flavonol glycosides, flavan-3-ols and derived proanthocyanidins
- **Table S10** Relative quantification of metabolites as determined by non-targeted UHPLC/ESI-QTOFMS analyses of methanolic *Ulmus minor* extracts

References

Supplementary Information: Protocols / Phytohormone extraction and measurement

Phytohormones were extracted based on a method described by Wang et al. (2007). In brief, 1 mL ethylacetate containing 2 µL of an internal standard mix (deuterated phytohormones 10 ng/µL D4-salicylic acid, 10 ng/µL D6-abscisic acid (OlChemIm Ltd., Olomouc, Czech Republic), 30.2 ng/µL D6-jasmonic acid and 10 ng/µL D6-jasmonyl-L-isoleucine (HPC Standards GmbH, Cunnersdorf, Germany) were added to the fine ground plant material together with Zirconium balls (Zirconox, 2.8-3.3 mm, Mühlmeier Mahltechnik, Bärnau, Germany), followed by 90 sec of shaking (FastPrep homogenizer, MP Biomedicals, Solon, OH, USA). Subsequently, the solution was centrifuged at 18,213 *g* and 4 °C (Eppendorf 5427 R centrifuge, Eppendorf AG, Hamburg, Deutschland) for 10 min and the supernatant transferred to a 2 mL Eppendorf tube. The extraction was repeated with 1 mL ethylacetate without internal standard, and afterwards supernatants were combined and evaporated at room temperature (RT) till a honey like drop remained (Eppendorf Concentrator 5301). The samples were re-dissolved in 400 µL 70 % methanol acidified with 0.1 % formic acid (v/v) on a Vortex for 10 min right before analysis. Samples were centrifuged for 15 min at 18,213 *g* at RT, and 200 µL of the particle-free supernatant were transferred to HPLC vials. Phytohormones were analysed by UPLC-ESI-MS/MS (Q-ToF-ESI) on a Synapt G2-S HDMS (Waters®, Milford, MA, USA). We injected 7 µL of each sample. Separation was achieved on a C_{18} column (Acquity UPLC Waters, BEH-C18, Ø 2.1 mm x 50 mm, particle size 1.7 µm). Water and methanol (each with 0.1 % formic acid (v/v)) were used as eluents A and B in a gradient mode with constant flow of 250 µL min^{-1} at 30 °C (eluent B: 0 min: 30 %; 1 min: 30 %; 4.5 min: 90 %; 8 min: 90 %; 9 min: 30 %; 3 min equilibration time between the runs). The separated compounds were electrospray-ionised at following conditions: capillary voltage 2.5 kV, nebulizer 6 bar, desolvation gas flow rate 500 L/h, 80 °C source temperature. Desolvation temperature was 150 °C, and N_2 was used as desolvation gas. Compounds were detected by tandem mass spectrometry scanning the full mass spectrum of compounds between 50–600 *m/z*.

Phytohormones were annotated according to their parent $[M\text{-}H]^-$ ion and a diagnostic daughter ion as well as according to co-elution with their deuterated derivatives (internal standard). Characteristic parent/daughter ions of the analysed phytohormones are: SA (*m/z* 137 and 93), ABA (*m/z* 263 and 153), JA (*m/z* 209 and 59), JA-Ile (*m/z* 322 and 130), and their deuterated derivatives: D4-SA (*m/z* 141 and 97), D6-ABA (*m/z* 269 and 159), for D6-JA (*m/z* 215 and 59), D6-JA-Ile (*m/z* 328 and 130). The phytohormones were quantified according to the peak area of the daughter ions of the plant-derived phytohormones relative to the daughter ions of the internal standards by using MassLynxTM Software (version 4.1; Waters). Concentrations per sample were normalised according to the fresh weight.

Supplementary Information: Protocols / RNA extraction

Total RNA was extracted from 50 mg of ground elm leaf material with 1.2 mL homogenisation buffer (0.2 M boric acid, 10 mM EDTA and 2 % SDS, adjusted to pH 7.6 with Tris base) to which ß-mercaptoethanol (5 %) and dithiothreitol (25 mM) had been freshly added. Samples were one by one mixed with extraction buffer in a Coolrack® (Corning Inc., Corning, NY, USA) on ice. The homogenisation step was followed by three steps of chloroform:isoamyl alcohol (C/I; 24:1, v/v) extraction. We added ca. 0.05 g of polyvinylpolypyrrolidone (PVPP) to the extraction step with the homogenisation buffer and to the first CI extraction step, thus reducing disturbance by polyphenols. After the third C/I step, disturbing polysaccharides were separated by extraction with ethanol (20 %), 560 mM potassium acetate and one volume of C/I. This step was followed by overnight precipitation at 4 °C with lithium chloride (LiCl, 2.67 M). After washing the pellet with 2 M cold LiCl and thereafter twice in cold 80 % ethanol, the pellet was dried, dissolved in nuclease-free water and frozen at -80 °C.

Supplementary Information: Protocols / HPLC-DAD settings for analyses of flavonol aglycones

HPLC-DAD analyses were performed on a Shimadzu HPLC system (Shimadzu Corp., Kyoto, Japan) comprising a degasser (DGU-20A3), a pump (LC-30AD), an auto injector (SIL-10A), a column thermostat (CO20, Torrey Pines Scientific, Carlsbad, CA, USA) and a diode array detector (SPD-M20A). Hydrolysed leaf extracts (injection volume 10 µL) were separated on an Intersil ODS-3 column (4.6 x 150 mm, 3 µm particle size, pre-column 4.6 x 15 mm, Intersil Corp., Milpitas, CA, USA) using 0.25 % phosphoric acid in water and acetonitrile as eluent A and B, respectively. The following binary gradient programme was used: 0-6 min, linear from 0 to 12 % B; 6-10 min, linear from 12 to 25 % B; 10-30 min, linear from 25 to 80 % B. A constant flow rate of 500 µL min^{-1} was used. The column temperature was maintained at 30 °C. Eluting compounds were monitored at 210, 254, 320 and 360 nm. Kaempferol (tR 19.92 min) and quercetin (tR 18.02 min) were quantified at 360 nm using external calibrations, which were established using authentic reference compounds (Sigma-Aldrich Corp., St. Louis, MO, USA).

Supplementary Information: Protocols / UHPLC/ESI-QTOFMS settings for untargeted analyses and quality control

Our analyses were performed on an Infinity 1290 series UHPLC system (Agilent Technologies Inc., Santa Clara, CA, USA) consisting of a binary pump (G4220A), an autosampler (G4226A, 20 µL loop), an autosampler thermostat (G1330B) and a thermostatted column compartment (G1316C), which was interfaced to an iFunnel Q-TOF mass spectrometer (G6550A, Agilent Technologies) via a dual Agilent

jet stream electrospray ion source. Extracts (1 µL injection volume) were separated on a Zorbax RRHD Eclipse Plus C_{18} column (100 × 2.1 mm, 1.8 µm particle size, Agilent Technologies) using 0.1 % (v/v) formic acid in water and 0.1 % (v/v) formic acid in acetonitrile as eluent A and B, respectively. The following binary gradient programme at a constant flow rate of 400 µL min^{-1} was applied: 0-12 min, linear from 5 % to 20 % B; 12-20 min, linear from 20 % to 50 % B; 20-23 min, isocratic, 95 % B; 23-25 min, isocratic, 5 % B. The column temperature was maintained at 40 °C and the autosampler temperature at 6 °C. The mass spectrometer was operated in low mass range and extended dynamic range (2 GHz) mode. Centroid mass spectra were acquired in negative ion mode from *m/z* 70-1200 using an acquisition rate of three spectra per second. The following instrument settings were applied: nebulizer gas, nitrogen, 35 psig; dry gas, nitrogen, 200 °C, 18 L min^{-1}; sheath gas, nitrogen, 300 °C, 12 L min^{-1}; capillary voltage, 3000 V; nozzle voltage, 0 V; high pressure funnel, voltage drop 200 V, RF voltage 150 V; low pressure funnel, voltage drop 100 V, RF voltage 100 V; funnel exit DC 50 V; octopole RF voltage, 750 V; collision gas, nitrogen; collision energy, 0 V. For reference mass correction a solution of purine (20 µM) and hexakis-(2,2,3,3-tetrafluoropropoxy)phosphazine (20 µM) in 95 % aqueous acetonitrile was continuously introduced through the second sprayer of the dual ion source at a flow rate of 20 µL min^{-1} using an external HPLC pump equipped with a 1:100 splitting device.

To monitor analytical performance, a pooled quality control (QC) sample was prepared by mixing 20 µL aliquots of each leaf extract. In addition, two blank extracts were prepared. The leaf and blank extracts were repeatedly analysed in random order. To check the quality of the obtained raw data, retention times and abundances of spiked internal standards were evaluated using MassHunter Quantitative Analysis software (Agilent Technologies).

Supplementary Information: Protocols / Pre-processing of untargeted UHPLC/ESI-QTOFMS data of semipolar compounds

Data pre-processing. Using MassHunter Qualitative Analysis software, raw data files were converted into mzData format using MassHunter Qualitative Analysis software, arranged in four sample classes (C, E, F, EF) and processed using the R package XCMS (Smith et al. 2006). Feature detection was performed using the centWave algorithm (Tautenhahn et al. 2008) [parameters: prefilter = (3, 1000); sntresh = 3; ppm = 25; peak width = (5, 12)]. Alignment was accomplished by consecutive application of the function group.density with two different parameter settings (parameters: minfrac = 1; bw = 2; mzwid = 0.02), retcor.loess (parameters: span = 1; missing = 1; extra = 1) and group.density (parameters: minfrac = 0.7; bw = 1.5, mzwid = 0.02). Alignment resulted in 3606 features. Missing

feature intensities were estimated using equally distributed random numbers in the interval [500,800]. Afterwards feature intensities were normalised by sample fresh weight and log_2 transformed.

Filter. To narrow down features of interest, we applied the following four filters. (I) We were mainly interested in compounds deriving from the phenylpropanoid pathway, which mainly consists of carbon-based, oxidized molecules. Due to the specific molecular weights of carbon, oxygen and hydrogen, precise molecular weights in the range of interest show a positive molecular mass shift. Therefore, we omitted features with mass shifts towards a negative weight, expressed via the first decimal place between 0.5 and 0.99. Crude plant extracts consist of many plant compounds, among them many salty or protein compounds. Usually, these compounds are either very polar or non-polar and reach the mass-spectrometer very early or towards the end of the chromatographic separation. Since these substances cause many misleading signals, we omitted features acquired during (II) the first 70 sec and (III) after 1000 sec from the results. (IV) Mean areas of features per treatment below an area of 13 (log_2 transformed) were often masked by matrix effects due to their low intensity. Consequently, we omitted features, which were below a log_2 transformed area of 13 in all four treatments. Applying all four filters, 1395 features were considered for further statistical analysis.

Supplementary Information: Protocols / Compound annotation by UHPLC/ESI-QTOFMS in targeted-MS/MS mode

For metabolite annotation, accurate mass collision-induced dissociation (CID) mass spectra were acquired by UHPLC/ESI-QTOFMS in targeted-MS/MS mode using scheduled precursor ion list with the following instrument parameters: acquisition rate MS, three spectra per second; acquisition rate MS/MS, 2 spectra per second, isolation width, narrow (1.3 *m/z*); collision energy, 10-60 V; collision gas, nitrogen (compare Supplementary Table S3 for MS/MS data, separate excel file). Based on accurate mass, isotope pattern and CID mass spectra, the putative elemental compositions were calculated for each compound using MassHunter Qualitative Analysis software. The obtained information was further used to query compound databases such as ChemSpider, PubChem and KEGG. In a next step, accurate mass CID mass spectra were matched against reference spectra collected in spectral libraries such as METLIN (Guijas et al. 2018) and MassBank (Horai et al. 2010). In case of no match, CID mass spectra were manually interpreted in order to refine or confirm initial hits from compound database or literature search. By this, 20 compounds could be identified to an annotation level of 1-4 according to Sumner et al. (2007). By comparison of chromatographic and mass spectral data, putative annotations of two metabolites (esculin, compound #22, Fig. 4, main text) and suberic acid (#31) were verified using commercially available reference compounds.

To comprehensively annotate *O*-glycosylated flavonols, sample analysis was conducted in positive ion mode with alternating collision energies of 0 V and 20 V (all ion fragmentation). Kaempferol, quercetin and isorhamnetin conjugates were detected via their protonated aglycones at *m/z* 287.055 (C15H11O6+), *m/z* 303.050 (C15H11O7+) and *m/z* 317.066 (C16H13O7+) at a collision energy of 20 V (Supplementary Fig. S1). Respective precursor ions of intact glycoconjugates were detected at the same retention time at a collision energy of 0 V and verified by analysis of respective data from negative ion mode. To validate the assignments and annotations obtained by all ion fragmentation, CID mass spectra were acquired in targeted-MS/MS targeted using protonated and deprotonated molecular ions as precursor ions (Supplementary Table S3). Identity of aglycones was confirmed by analysis of pseudo-MS3 spectra obtained from protonated aglycone ions, whose formation was induced by in-source fragmentation (funnel exit DC 140 V). The obtained spectra were referenced against CID mass spectra obtained from $[M+H]^+$ ions of authentic kaempferol (K), quercetin (Q) and isorhamnetin (I). Among the compounds shown in Fig. 3, main text, identities of K-3-Rut (compound #7), K-3-Glc (#8), Q-3-Rut (#14), Q-3-GlcA (#15), Q-3-Glc (#16) and I-3-Rut (#18) were verified by comparison of chromatographic and mass spectral data obtained from commercially available reference compounds. Flavan-3-ols and derived dimeric and trimeric proanthocyanidins were annotated based on accurate tandem mass spectral data. Identity of catechin and epicatechin were verified by authentic reference compounds.

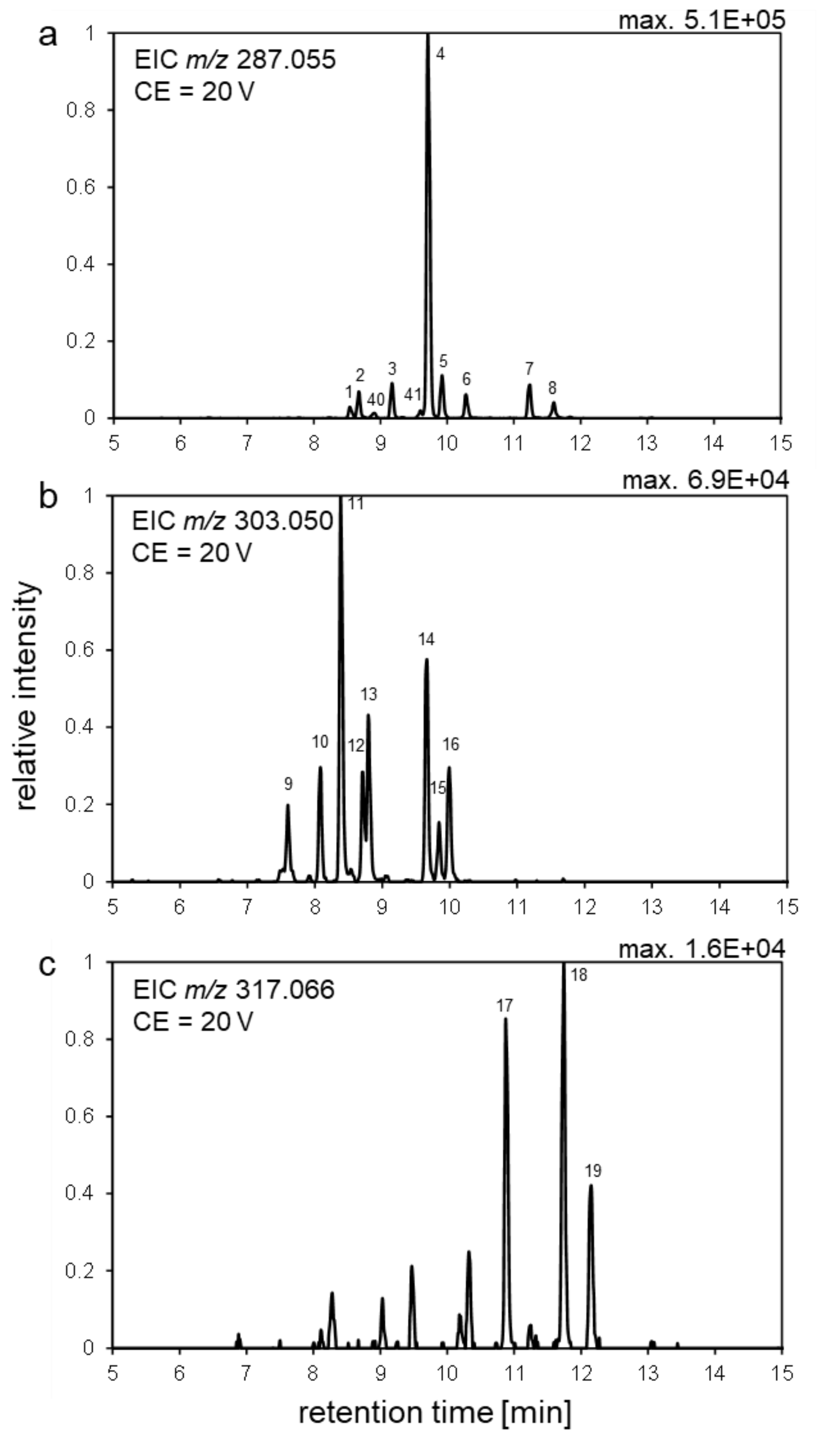

Fig. S1 Detection of *O*-glycosylated flavonols via their protonated aglycone using all-ion fragmentation. The extracted ion chromatograms (EIC) corresponding to kaempferol *O*-glycosides (1-8, 40, 41) is shown in **a**, EICs corresponding to quercetin *O*-glycosides (9-16) and isorhamnetin *O*-glycosides (17-19) in **b** and **c**, respectively. Compound numbers refer to Supplementary Table S3. Chromatograms were obtained from a pooled methanolic elm leaf extract using reversed-phase UHPLC/ESI-QTOFMS in positive ion mode at a collision energy of 20 V. The pooled elm leaf extract was prepared by mixing equal aliquots of individual elm leaf extracts of either treatment (after 24 h larval feeding or at an equivalent time point for treatments without larval feeding)

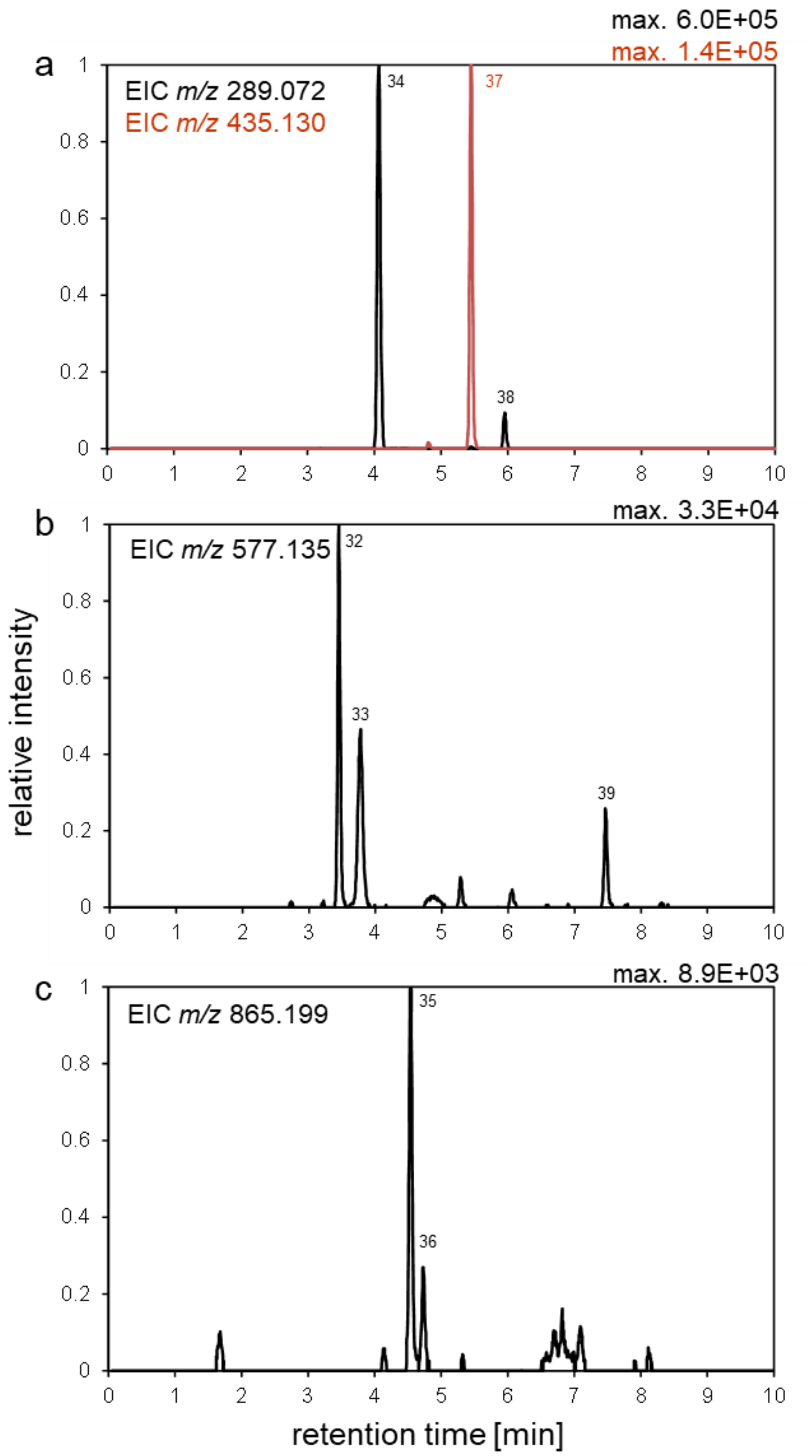

Fig. S2 Detection of flavan-3-ols (catechins, epicatechins) and derived dimeric and trimeric proanthocyanidins via their deprotonated molecular ions. Extracted ion chromatograms (EICs) corresponding to catechin (34) and epicatechin (38), as well as to an (epi)catechin *O*-deoxyhexoside (37), are shown in **a**. EICs corresponding to dimeric (32-33, 39) and trimeric proanthocyanidins (35-36) derived from catechin and/or epicatechin are shown in **b** and **c**, respectively. Compound numbers refer to Supplementary Table S3. Chromatograms were obtained from a pooled methanolic elm leaf extract using reversed-phase UHPLC/ESI-QTOFMS in negative ion mode. The pooled elm leaf extract was prepared by mixing equal aliquots of individual elm leaf extracts of either treatment (after 24 h larval feeding or at an equivalent time point for treatments without larval feeding)

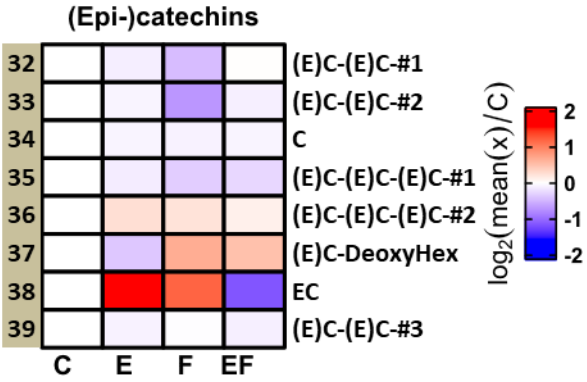

Fig. S3 Impact of elm leaf beetle egg deposition and larval feeding on *Ulmus minor* (epi-)catechin contents. Heatmap shows log_2 fold change of metabolite levels in methanolic leaf extracts by UHPLC/ESI-QTOFMS – analysis. Left side of heatmap: Compound numbers refer to Supplementary Table S3. Right side: abbreviations of detected compounds, (E)C = (Epi)catechin, C = Catechin, EC = Epicatechin, DeoxyHex=Deoxyhexose; Bottom of heatmap: C = untreated control leaves, E = locally egg-treated leaves, F = locally feeding-damaged leaves, 24 h feeding period, EF = locally egg-treated, feeding-damaged leaves, 24 h feeding period. Log_2 fold change relative to control was calculated by log_2 of the ratio of mean peak area per metabolite in a treated leaf relative to the mean of the respective metabolite peak area in the control. Statistics: ANOVA did not show significant differences at $P<0.05$; $n=9-10$

Table S1 Primers for putative *Ulmus minor* genes involved in the biosynthesis of phenylpropanoids

Gene[a]	Contig[b]	forward/reverse primer sequence (5'-3')	forward/reverse primer name	Amplicon size (bp)
PAL Phenylalanine ammonia-lyase	C598923	GCGAGCAGCGATTGGGTTATG/ CCTTGTTTGGTTCGCCTGTGG	259-elm_03692_PAL_F/ 260-elm_03692_PAL_R	101
4CL 4-coumarate ligase-like 9	C608925	AATACAAGGCGTATCAGGTTCCG/ TTCCAGGCTTTCTCACCACATAG	265-elm_11002_4CL_9_F/ 266- elm_11002_4CL_9_R	144
HCT Shikimate *O*-hydroxycinnamoyl-transferase-like	scaffold23443	AGAGATCCACCTCGaCCCAA/ TTTGAACATGGCAACCGCAG	333-elm_12392_New_SOHC_F/ 334-elm_12392_New_SOHC_R	111
CAD Cinnamyl alcohol dehydrogenase	scaffold16882	ACAGATGAAGGCCCTGGCTAAG/ TCGTGCCACCAACTACACTACC	267-elm_07576_CAD_F/ 268-elm_07576_CAD_R	200
COMT/ Caffeic acid 3-*O*-methyltransferase-like	scaffold7507	TAGCCGATTGGTTGATGTTG/ TGGCATCACCATTTGGTAAG	331-elm_13443_New_CA_3OMT_F/ 332-elm_13443_New_CA_3OMT_R	182
FLS/F3H Flavonol synthase/ flavanone 3-hydroxylase-like	scaffold5562	TCCTTAGCCGAATCACCCAAC/ CCAGAGGTGAGAAGGGAGAAATC	247-elm_09910_FSF_3H_F/ 248-elm_09910_FSF_3H_R	131
F3'H Flavonoid 3'-monooxygenase-like	C607209	CAAATCGCAGGCTACGACATTC/ GCTCAAAGTCTTGGCCTTTCAC	285-elm_23041_F31Ox_F/ 286-elm_23041_F31Ox_R	154
ANS Leucoanthocyanidin dioxygenase-like	scaffold3763	ATTCGGATCCTGGTGGGATGAC/ CGTGTGGAGCAGGTTTAACAGTG	241-elm_05108_LA_2Ox_F/ 242-elm_05108_LA_2Ox_R	105

[a] Annotation according to Altmann et al. (2018) derived from a mapping of the elm transcriptome against the *Arabidopsis* transcriptome (TAIR10 annotation), and from Perdiguero et al. (2015), based on blast queries (BlastN, threshold E < 10–6).
[b] Contigs from TSA (Transcriptome shotgun assembly) BioProjectID 312302 (Altmann et al. 2018)

Table S2 Reference genes and their primer sequences

Gene	Amplicon size (bp)	Primer name	forward/reverse primer sequence (5'-3')
Ubiquitin	158	5/elm_00177_Ubi_F<br>6/elm_00177_Ubi_R	GACAACGTGAAGGCGAAAATCCA<br>CCACCCCTGAGACGAAGCACA
SAND family gene	97	12/Um18663-SAND_F3<br>13/Um18663-SAND_R2	CAGCAGGACCACCTACACCA<br>TCGCACTGGATCATCACCTC
splicing factor3B subunit 5-like protein	119	97/elm_splicing3B-SU_F<br>98/elm_splicing3B-SU_R	TGCTTTTGCCTTGCGGTCTT<br>CAGTGCTTCAACCAACCGGATTT

Table S3 Not in this document; see separate supplementary file 2 (Supplementary Table S3 MS_MS analytical data)

Table S4 Phytohormone levels in locally treated *Ulmus minor* leaves after 24 h of larval feeding and at an equivalent time point for treatments without larval feeding. C = untreated control leaves, E = egg-treated leaves, F = feeding-damaged leaves, EF = egg-treated, feeding-damaged leaves. Concentration in ng/g FW as mean ± SE, *n*=9-10. ANOVA + Tukey of log-transformed data, *P*<0.05. Different letters below means ± SE indicate significant differences between treatments

Phyto-hormone[a]	Time point	Treatment C	E	F	EF	ANOVA, *P* value
**SA**	6h	493 ±79	426 ±72	355 ±87	282 ±43	0.159
	**24h**	**449 ±85 a**	**719 ±99 a**	**704 ±118 a**	**1365 ±85 b**	**<0.001**
**JA**	**6h**	**0.9 ±0.2 a**	**1.3 ±0.3 a**	**104.0 ±19.4 b**	**84.1 ±9.4 b**	**<0.001**
	**24h**	**0.6 ±0.1 a**	**1.1 ±0.2 a**	**153.7 ±29.0 b**	**162.7 ±33.4 b**	**<0.001**
**JA-Ile**	**6h**	**2.1 ±0.6 a**	**2.1 ±0.3 a**	**27.8 ±3.6 b**	**29.0 ±2.7 b**	**<0.001**
	**24h**	**0.9 ±0.1 a**	**1.1 ±0.2 a**	**80.9 ±19.3 b**	**62.9 ±12.9 b**	**<0.001**
ABA	6h	317 ±32	261 ±26	225 ±35	294 ±52	0.296
	24h	361 ±49	470 ±53	355 ±47	382 ±19	0.226

[a] SA = salicylic acid, JA = jasmonic acid, JA-Ile = jasmonic acid–isoleucine, ABA = abscisic acid

Table S5 Phytohormone levels in *Ulmus minor* leaves adjacent to treated leaves ('systemic' leaves) after 24 h of larval feeding and at an equivalent time point for treatments without larval feeding. C = untreated control leaves, E = egg-treated leaves, F = feeding-damaged leaves, EF = egg-treated, feeding-damaged leaves. Concentration in ng/g FW as mean ± SE, *n*=9-10. ANOVA + Tukey of log-transformed data, *P*<0.05. Different letters below means ± SE indicate significant differences between treatments

Phyto-hormone[a]	Time point	Treatment C	E	F	EF	ANOVA, *P* value
SA	6h	1684 ±184	1465 ±187	1765 ±279	1334 ±181	0.582
	24h	299 ±50	385 ±74	478 ±136	373 ±47	0.623
**JA**	**6h**	**1.9 ±0.7 ab**	**1.2 ±0.3 a**	**2.8 ±0.7 b**	**2.0 ±0.3 ab**	**0.034**
	**24h**	**1.2 ±0.2 a**	**1.3 ±0.2 a**	**1.6 ±0.2 ab**	**2.3 ±0.4 b**	**0.028**
**JA-Ile**	6h	1.7 ±0.5	1.5 ±0.1	2.1 ±0.4	2.3 ±0.3	0.134
	**24h**	**1.3 ±0.1 a**	**1.4 ±0.2 a**	**2.8 ±0.4 ab**	**2.9 ±0.5 b**	**<0.001**
ABA	6h	538 ±64	472 ±43	451 ±65	618 ±81	0.351
	24h	330 ±43	297 ±38	218 ±31	268 ±22	0.091

[a] SA = salicylic acid, JA = jasmonic acid, JA-Ile = jasmonic acid–isoleucine, ABA = abscisic acid

Table S6 Expression of homologues of phenylpropanoid synthesis-related genes in local *Ulmus minor* leaves. Gene expression was measured in treated leaves after 6 h or 24 h of larval feeding and at an equivalent time point for treatments without larval feeding. C = untreated control leaves, E = egg-treated leaves, F = feeding-damaged leaves, EF = egg-treated, feeding-damaged leaves. Medians (25 %, 75 % quartiles) of relative expression (fold change normalised to C) are shown. Different letters next to medians indicate significant differences between treatments. Kruskal-Wallis test + Wilcoxon rank-sum test, $P<0.05$; n=7-10

Gene[a]	Time point	Treatment C	E	F	EF	Kruskal-Wallis test, *P*-value
PAL	**6h**	**0.89 a** (0.81, 1.32)	**1.11 a** (0.99, 1.26)	**3.86 b** (3.67, 5.51)	**5.07 b** (3.64, 6.03)	**< 0.001**
	**24h**	**0.83 a** (0.70, 0.89)	**0.63 a** (0.40, 1.35)	**5.45 b** (3.99, 5.77)	**7.57 c** (5.92, 9.79)	**< 0.001**
4CL	6h	0.97 (0.90, 1.21)	1.26 (0.90, 1.55)	0.93 (0.87, 1.16)	1.07 (0.80, 1.42)	0.629
	24h	1.04 (0.87, 1.12)	0.94 (0.84, 1.11)	1.00 (0.95, 1.07)	0.76 (0.67, 0.91)	0.102
HCT	6h	**1.02 ab** (0.90, 1.14)	**0.83 a** (0.79, 0.93)	**1.26 b** (0.94, 1.57)	**1.26 b** (1.05, 1.85)	**0.013**
	24h	0.87 (0.74, 0.98)	0.83 (0.58, 1.25)	1.22 (0.88, 1.92)	1.05 (0.92, 1.62)	0.211
CAD	6h	0.99 (0.86, 1.09)	1.01 (0.64, 1.10)	0.84 (0.75, 1.04)	1.00 (0.82, 1.16)	0.552
	24h	1.04 (0.78, 1.18)	1.20 (1.02, 1.45)	1.14 (1.02, 1.30)	1.35 (1.03, 1.39)	0.347
COMT	6h	1.02 (0.86, 1.30)	1.21 (0.67, 1.55)	1.20 (0.86, 1.41)	0.68 (0.51, 0.77)	0.093
	24h	1.06 (0.76, 1.30)	1.04 (0.85, 1.23)	0.87 (0.72, 1.18	0.55 (0.53, 0.83)	0.116
FLS/F3H	6h	0.84 (0.77, 1.08)	0.96 (0.81, 1.05)	0.59 (0.48, 0.80)	0.66 (0.56, 0.86)	**0.018**[b]
	24h	1.03 (0.84, 1.21)	0.98 (0.84, 1.23)	0.78 (0.65, 0.92)	0.63 (0.51, 0.84)	**0.036**[b]
F3'H	6h	1.06 (0.77, 1.27)	1.06 (0.73, 1.14)	1.18 (0.70, 1.38)	1.18 (0.86, 1.69)	0.645
	24h	1.09 (0.77, 1.25)	0.95 (0.54, 1.17)	1.22 (1.14, 2.29)	1.08 (1.01, 1.23)	0.213
ANS	**6h**	**0.96 a** (0.80, 1.25)	**1.01 a** (0.92, 1.26)	**3.58 b** (3.12, 4.65)	**3.64 b** (3.32, 4.50)	**< 0.001**
	**24h**	**0.80 a** (0.60, 0.97)	**0.71 a** (0.56, 1.17)	**7.05 b** (6.46, 9.19)	**5.84 b** (5.26, 6.83)	**< 0.001**

[a] Compare Supplementary Table S1 for full names of genes
[b] Kruskal-Wallis test significant, but Wilcoxon rank sum test not significant

Table S7 Expression of homologues of phenylpropanoid synthesis-related genes in systemic *Ulmus minor* leaves. Gene expression was measured in leaves adjacent to treated leaves after 24 h of larval feeding and at an equivalent time point for treatments without larval feeding. C = untreated control leaves, E = egg-treated leaves, F = feeding-damaged leaves, EF = egg-treated, feeding-damaged leaves. Medians (25 %, 75 % quartiles) of relative expression (fold change, normalised to C) are shown. Different letters next to medians indicate significant differences between treatments. Kruskal-Wallis test + Wilcoxon rank-sum test, $P<0.05$; n=7-10

Gene[a]	Time point	Treatment C	E	F	EF	Kruskal-Wallis test, *P* value
PAL	**24h**	**0.87 a** (0.74, 1.08)	**1.29 ab** (0.67, 1.74)	**2.14 b** (1.44, 3.75)	**1.50 b** (1.32, 2.15)	**0.006**
4CL	24h	1.09 (0.77, 1.16)	1.09 (1.05, 1.32)	1.19 (1.14, 1.35)	0.96 (0.87, 1.07)	0.068
HCT	24h	1.02 (0.83, 1.32)	0.94 (0.88, 1.27)	1.02 (0.94, 1.54)	1.05 (0.79, 1.28)	0.918
CAD	24h	0.99 (0.80, 1.19)	1.36 (1.19, 1.60)	1.41 (1.03, 1.50)	0.99 (0.87, 1.41)	0.331
COMT	24h	0.99 (0.66, 1.51)	0.96 (0.75, 1.13)	0.94 (0.62, 1.41)	0.93 (0.58, 1.09)	0.828
FLS/F3H	24h	1.21 (0.85, 1.31)	1.21 (1.15, 1.37)	1.14 (1.02, 1.22)	0.90 (0.83, 1.06)	0.0637
F3'H	24h	0.89 (0.74, 1.19)	1.17 (0.50, 1.61)	1.08 (0.97, 1.15)	0.81 (0.59, 0.97)	0.516
ANS	**24h**	**0.87 a** (0.63, 0.94)	**0.98 a** (0.71, 1.35)	**2.75 b** (2.18, 3.58)	**1.61 b** (1.43, 2.44)	**<0.001**

[a] Compare Supplementary Table S1 for full names of genes

Table S8 Concentrations of kaempferol and quercetin aglycones derived from acidified (hydrolysed) *Ulmus minor* extracts. HPLC-DAD analysis of acid-hydrolysed methanolic leaf extracts. C = untreated control leaves, E = egg-treated leaves, F = locally feeding-damaged leaves, 24 h feeding period, EF = locally egg-treated, feeding-damaged leaves, 24 h feeding period. Concentration in µg/g FW as mean ± SE, n=9-10. Different letters below means ± SE indicate significant differences between treatments. ANOVA + Tukey, $P<0.05$

Compound	Treatment C	E	F	EF	ANOVA, *P* value
**Kaempferol**	**22.5 ± 6.1 a**	**22.9 ± 3.9 a**	**27.6 ± 4.8 ab**	**54.2 ± 10.2 b**	**0.009**
**Quercetin**	**10.8 ± 3.0 a**	**19.5 ± 2.1 ab**	**14.2 ± 1.1 a**	**27.6 ± 3.6 b**	**0.001**

Table S9 Relative quantification of flavonol glycosides, flavan-3-ols and derived proanthocyanidins. Metabolites detected in methanolic leaf extracts by UHPLC/-ESI-QTOFMS analysis. C = untreated control leaves, E = egg-treated leaves, F = locally feeding-damaged leaves, 24 h feeding period, EF = locally egg-treated, feeding-damaged leaves, 24 h feeding period. Mean peak area ± SD, *n*=9-10, different letters below means ± SD indicate significant differences between treatments at *P*<0.05 (Tukey post-hoc test); ANOVA *P*-values: see this table. For compounds #20-30 and #31, see Table S10 and Table S3, respectively

No.[a]	Compound[b]	Treatment				ANOVA, *P* value
		C	E	F	EF	
1	K-Hex-Pent-DeoxyHex-#1	19019 ± 33844	11287 ± 20150	13104 ± 9095	145572 ± 248241	0.086
2	K-Hex-Pent-DeoxyHex-#2	55821 ± 81048	47016 ± 45568	72575 ± 47661	151763 ± 155669	0.338
3	K-Hex-(DeoxyHex)2-#2	65353 ± 64259	42382 ± 24844	134518 ± 116951	243183 ± 350672	0.241
4	K-HexA-DeoxyHex	1043055 ± 1410172	678768 ± 576092	1808965 ± 914983	1481657 ± 1389023	0.502
**5**	**K-Hex-DeoxyHex-#2**	**66851 ± 67169**<br>**a**	**95531 ± 93595**<br>**ab**	**147417 ± 92677**<br>**ab**	**377409 ± 505493**<br>**b**	**0.036**
6	K-Hex-Pent	75922 ± 70987	87562 ± 64958	82685 ± 58251	119282 ± 116005	0.857
7	K-3-Rut	170737 ± 93497	192111 ± 94035	271725 ± 179852	283119 ± 223806	0.598
8	K-3-Glc	87679 ± 57702	108125 ± 60931	129238 ± 80757	149558 ± 123296	0.836
9	Q-Hex-Pent-DeoxyHex-#1	21922 ± 20017	27937 ± 14942	29261 ± 14551	27771 ± 21570	0.554
10	Q-Hex-(DeoxyHex)2-#1	25946 ± 19807	28798 ± 14198	54612 ± 32534	55440 ± 65610	0.231

No.[a]	Compound[b]	Treatment C	E	F	EF	ANOVA, *P* value
11	Q-HexA-DeoxyHex	216592 ± 373838	135919 ± 81053	336188 ± 288238	219383 ± 259300	0.368
12	Q-Hex-DeoxyHex	30183 ± 22353	50267 ± 38264	63068 ± 38331	80007 ± 67552	0.069
13	Q-Hex-Pent	61614 ± 43471	82628 ± 81060	81030 ± 59571	124440 ± 178579	0.838
14	Q-3-Rut	226528 ± 172445	191444 ± 85283	336442 ± 143643	391880 ± 494668	0.513
15	Q-3-GlcA	15439 ± 15904	37274 ± 68741	31982 ± 28585	55479 ± 96762	0.469
16	Q-3-Glc	73106 ± 43471	93943 ± 75245	125141 ± 76540	155820 ± 172156	0.440
17	I-Hex-Pent	47796 ± 42210	28964 ± 33367	28499 ± 21949	18960 ± 11371	0.595
18	I-3-Rut	98089 ± 99889	50649 ± 36133	67364 ± 53453	47127 ± 31960	0.890
19	I-Hex	50296 ± 41895	37520 ± 17455	39442 ± 29622	37281 ± 20087	0.936
32	(E)C-(E)C-#1	122215 ± 132153	113212 ± 120980	90801 ± 80691	123048 ± 175371	0.911
33	(E)C-(E)C-#2	112523 ± 156719	107296 ± 115784	70272 ± 56117	104864 ± 152238	0.930
34	C	2041981 ± 2007721	1945041 ± 1512416	1929116 ± 1645731	1945396 ± 1658832	0.961

No.[a]	Compound[b]	Treatment<br>C	 E	 F	 EF	ANOVA, *P* value
35	(E)C-(E)C-(E)C-#1	38782 ± 47145	35631 ± 38904	31112 ± 39342	32509 ± 44598	0.929
36	(E)C-(E)C-(E)C-#2	8129 ± 7622	9667 ± 8980	9478 ± 10289	8799 ± 11724	0.787
37	(E)C-DeoxyHex	346312 ± 260881	270060 ± 316813	538970 ± 427097	484547 ± 612457	0.621
38	EC	113047 ± 113727	425507 ± 643033	250371 ± 552088	52738 ± 41734	0.297
39	(E)C-(E)C-#3	33176 ± 36354	31387 ± 27743	32948 ± 41622	30876 ± 39577	0.927

[a] Two kaempferol derivatives (compound No. 40 and 41, see Supplementary Table S3) were found in targeted analysis but were excluded from further statistical analysis due to a high number of measurements below detection limit

[b] For explanation of compound abbreviations please refer to Supplementary Table S3

Table S10 Relative quantification of phenylpropanoid metabolites as determined by non-targeted UHPLC/ESI-QTOFMS analyses of methanolic *Ulmus minor* extracts. C = untreated control leaves, E = egg-treated leaves, F = locally feeding-damaged leaves, 24 h feeding period, EF = locally egg-treated, feeding-damaged leaves, 24 h feeding period. Mean peak area ± SD, *n*=9-10, different letters below means ± SD indicate significant differences between treatments at *P*<0.05 (Tukey post-hoc test)

No.	Compound[a]	Treatment				ANOVA, *P*-value
		C	E	F	EF	
20	Api-C-Pent-C-Hex	489677 ± 377814	160704 ± 127836	185685 ± 131930	306571 ± 412482	0.122
21	Api-di-C-Hex	199699 ± 108692	126129 ± 107074	88927 ± 65571	136550 ± 142346	0.251
22	Esculin	121274 ± 45751	201781 ± 117121	262947 ± 103973	254213 ± 165385	0.068
23	Tyrosol-Hex	40882 ± 33891	92606 ± 82701	50874 ± 33742	62466 ± 22724	0.065
**24**	**Coum-Hex-#1**	**70186 ± 29804**<br>**a**	**118169 ± 101588**<br>**A**	**577322 ± 280090**<br>**b**	**463589 ± 275953**<br>**b**	**<0.001**
**25**	**Coum-Hex-#2**	**5653 ± 5943**<br>**a**	**11356 ± 17972**<br>**A**	**61784 ± 30341**<br>**b**	**46781 ± 35740**<br>**b**	**<0.001**
**26**	**Coum-Quinate**	**212393 ± 308830**<br>**a**	**366906 ± 301008**<br>**Ab**	**433997 ± 369719**<br>**ab**	**651451 ± 520902**<br>**b**	**0.017**
27	Syringylglycerol-Hex	48897 ± 30007	48288 ± 24550	99315 ± 51150.	65626 ± 42148	0.093
28	Pinoresinol-Hex	10804 ± 11224	56564 ± 80918	8922 ± 5049	17448 ± 27780	0.074
29	Lariciresinol-Hex	155384 ± 206585	326974 ± 136046	170963 ± 197902	174356 ± 196795	0.065
30	Justiciresinol-Hex	12516 ± 7179	23071 ± 12095	23400 ± 19867	19313 ± 11713	0.409

[a] For explanation of compound abbreviations please refer to Supplementary Table S3

.

References

Altmann S, Muino JM, Lortzing V, Brandt R, Himmelbach A, Altschmied L, Hilker M (2018) Transcriptomic basis for reinforcement of elm antiherbivore defence mediated by insect egg deposition. Mol Ecol 27:4901–4915. https://doi.org/10.1111/mec.14900

Guijas C, Montenegro-Burke JR, Domingo-Almenara X, Palermo A, Warth B, Hermann G, Koellensperger G, Huan T, Uritboonthai W, Aisporna AE, Wolan DW, Spilker ME, Benton HP, Siuzdak G (2018) METLIN: A technology platform for identifying knowns and unknowns. Anal Chem 90:3156–3164. https://doi.org/10.1021/acs.analchem.7b04424

Horai H, Arita M, Kanaya S, et al (2010) MassBank: a public repository for sharing mass spectral data for life sciences. J Mass Spectrom 45:703–714. https://doi.org/10.1002/jms.1777

Perdiguero P, Venturas M, Cervera MT, Gil L, Collada C (2015) Massive sequencing of *Ulmus minor's* transcriptome provides new molecular tools for a genus under the constant threat of Dutch elm disease. Front Plant Sci 6:541. https://doi.org/10.3389/fpls.2015.00541

Smith CA, Want EJ, O'Maille G, Abagyan R, Siuzdak G (2006) XCMS: processing mass spectrometry data for metabolite profiling using nonlinear peak alignment, matching, and identification. Anal Chem 78:779–787. https://doi.org/10.1021/ac051437y

Sumner LW, Amberg A, Barrett D, et al (2007) Proposed minimum reporting standards for chemical analysis Chemical Analysis Working Group (CAWG) Metabolomics Standards Initiative (MSI). Metabolomics 3:211–221. https://doi.org/10.1007/s11306-007-0082-2

Tautenhahn R, Böttcher C, Neumann S (2008) Highly sensitive feature detection for high resolution LC/MS. BMC Bioinform 9:504. https://doi.org/10.1186/1471-2105-9-504

Wang D, Pajerowska-Mukhtar K, Culler AH, Dong X (2007) Salicylic acid inhibits pathogen growth in plants through repression of the auxin signaling pathway. Curr Biol 17:1784–1790. https://doi.org/10.1016/j.cub.2007.09.025
